# An integrative variant analysis suite for whole exome next-generation sequencing data

**DOI:** 10.1186/1471-2105-13-8

**Published:** 2012-01-12

**Authors:** Danny Challis, Jin Yu, Uday S Evani, Andrew R Jackson, Sameer Paithankar, Cristian Coarfa, Aleksandar Milosavljevic, Richard A Gibbs, Fuli Yu

**Affiliations:** 1 The Human Genome Sequencing Center, Baylor College of Medicine, Houston, USA; 2 Department of Molecular and Human Genetics, Baylor College of Medicine, Houston, USA

## Abstract

**Background:**

Whole exome capture sequencing allows researchers to cost-effectively sequence the coding regions of the genome. Although the exome capture sequencing methods have become routine and well established, there is currently a lack of tools specialized for variant calling in this type of data.

**Results:**

Using statistical models trained on validated whole-exome capture sequencing data, the Atlas2 Suite is an integrative variant analysis pipeline optimized for variant discovery on all three of the widely used next generation sequencing platforms (SOLiD, Illumina, and Roche 454). The suite employs logistic regression models in conjunction with user-adjustable cutoffs to accurately separate true SNPs and INDELs from sequencing and mapping errors with high sensitivity (96.7%).

**Conclusion:**

We have implemented the Atlas2 Suite and applied it to 92 whole exome samples from the 1000 Genomes Project. The Atlas2 Suite is available for download at http://sourceforge.net/projects/atlas2/. In addition to a command line version, the suite has been integrated into the Genboree Workbench, allowing biomedical scientists with minimal informatics expertise to remotely call, view, and further analyze variants through a simple web interface. The existing genomic databases displayed via the Genboree browser also streamline the process from variant discovery to functional genomics analysis, resulting in an off-the-shelf toolkit for the broader community.

## Background

Whole Exome Capture Sequencing (WECS) represents a cost effective approach to identify the mutations of highest biomedical importance, generating hypotheses for downstream follow-up [1]. The cost of WECS is currently only ~1/10 of the cost of whole genome sequencing performed on next-generation sequencing (NGS) platforms; it is a flexible approach designed to detect rare variants segregating in affected families, to follow up on already identified regions of interest in large scale association studies, and to produce more interpretable variant results [2-4]. In the past few years, WECS has rapidly gained popularity in disease studies and has even helped reveal many highly interesting causal loci [5,6]

While capture sequencing technologies have matured and stabilized to a level appropriate for routine use, there are few generally available analysis tools specialized for dealing with exome capture data. WECS data introduces a set of biases and error patterns distinct from whole genome sequencing, such as heterogeneous depth coverage, and reference bias due to capture [7]. The coding regions of the human genome also modify the expectation of a number of quality metrics that are routinely used in variant calling such as Transition/Transversion ratio (Ts/Tv), and percentage of insertion-deletion polymorphisms introducing frameshifts. Thus far, a few bioinformatics tools primarily developed for whole genome analysis have been utilized in WECS with some success, but extensive manual adjustments--such as setting stricter cutoffs and/or implementing several additional ad-hoc filters pertinent to coding regions--based on the user's own biomedical and informatic insights are required [8,9].

Here we present the Atlas2 Suite, a suite of tools specialized for calling variants in WECS on multiple sequencing platforms (i.e. SOLiD, Roche 454, and Illumina) which detects both SNPs and short range (within tens of base pairs) insertion-deletions (INDELs). (For the purposes of this study we describe the model specialized for SOLiD data in the main text. Details on the Illumina and Roche 454 SNP models can be found in our previous publication [10], and the Illumina INDEL model is described in the Supplement (Additional file 1)). The Atlas2 Suite makes use of logistic regression models trained on WECS data to identify SNP and INDEL sites with high sensitivity and specificity, and to subsequently produce genotypes.

**Figure 1:**
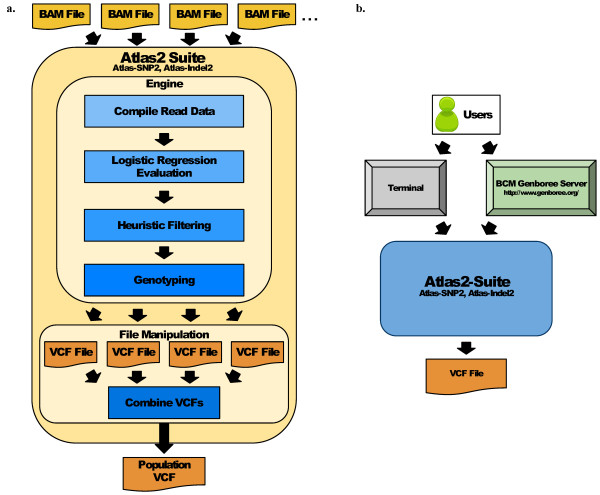
**The Atlas2 Suite Pipeline**. (a) The Atlas2 Suite is designed to accept as input single sample BAM files which are individually processed by Atlas-SNP2 and/or Atlas-Indel2 to produce single sample variant calls in VCF format. Both Atlas-SNP2 and Atlas-Indel2 use the same basic algorithm: for each variant site all the read data is compiled, the compiled data is fed into a logistic regression model for evaluation, and variants that are of sufficiently high quality and pass the heuristic filters are then genotyped and output as a VCF file. For population analysis, multiple single-sample VCF files may be combined into a population-level VCF with any missing coverage information filled in. (b) The Atlas2 Suite is available for use in both a command line version and through the Baylor College of Medicine (BCM) Genboree Server.

Wide adoption of WECS in both research and clinical settings is currently limited by the data analysis bottleneck including the shortage of bioinformatics expertise and inadequate computational resources. Small clinical laboratories are particularly affected by this limitation. To address this bottleneck, we made the Atlas2 Suite web-accessible via the Genboree Discovery System http://www.genboree.org (Figure 1 and Additional file 1, Figure S7), allowing researchers with a minimal level of bioinformatics training and computational resources, using only a web browser to carry out variant analysis, understand functional implications, and generate hypotheses for followup studies. In the context of a research study, the additional access control, role assignment, data protection, and collaborative features of Genboree aids researchers in meeting many administrative, physical, and technical provisions of the HIPAA Privacy Rule.

## Implementation

### Overview of Atlas2 Pipeline

The primary components of the Atlas2 Suite are Atlas-SNP2 and Atlas-Indel2 (Figure 1). Each of these applications accepts single sample alignment data in Binary Sequence Alignment/Mapping format (BAM) [8] and produces SNP or INDEL calls in the Variant Call Format (VCF) [11]. The initial single sample VCF files may be merged and annotated into a population VCF file using the 'vcfPrinter' module within Atlas2.

To separate true variants from sequencing, mapping, and alignment errors, the Atlas2 Suite collects read depths for each allele, read quality scores, and other pertinent data at each variant locus and applies a trained logistic regression model to assess the quality of each potential variant (Figure 1a, Table 1). We exhaustively examined possible variables (Additional file 1, Tables S1 and S2 and Figures S2 and S5), and found the most significant variables were related to read depth ratio, base quality score, variant position in the read, and the read strand direction distribution. The logistic regression models return the probability (*p*) that the given variant is a true variant. To deal with the small number of edge cases which the model may misclassify, and for another layer of flexibility on the users' end, variant calls are subjected to a few basic heuristic filters such as minimum read depth and minimum variant read ratio. Variants with a *p *greater than a default cutoff and which pass the heuristic filters are included in the final variant call set in VCF format. Based on our initial evaluation results, we optimized the suite's cutoffs and options to balance sensitivity and precision. Users are also able to tune the parameters to make a call set more suitable to their data and research context.

**Table 1:** Atlas2 Suite SOLiD model variables

SNPs			
**Description**	**Type**	**z-value**	**p-value**

Reference/variant reads ratio	Numeric	-34.36	< 2E-16

Strand direction	Boolean	17.57	< 2E-16

Mean distance to 3' end	Numeric	16.87	< 2E-16

Mean neighboring base quality (NBQ)	Numeric	14.83	< 2E-16

Mean variant base quality	Numeric	10.61	< 2E-16

Mean NBQ × Mean distance 3' (interaction)	Numeric	-12.39	< 2E-16

Strand direction × Mean distance 3' (interaction)	Numeric	-10.52	< 2E-16

**INDELs**			

**Description**	**Type**	**z-value**	**p-value**

Mean neighbor base quality (NBQ)	Numeric	9.097	< 2E-16

Normalized variant square (NVS)	Numeric	7.035	2.00E-12

Mean variation rate	Numeric	-6.669	2.57E-11

Read end ratio	Numeric	-4.436	9.15E-06

Variables and interactions for both the SNP and INDEL models along with the variable's Wald z-statistic and p-value as estimated by the R environment's glm function. The z-values and p-values indicate the significance of the variables in the model, with the most significant variables having a z-value furthest from zero.

The Atlas2 Suite is currently specialized for variant detection within exonic regions on existing NGS platforms, but it also can easily be evolved to work with new technologies. In order to identify systematic sequencing and mapping errors on a wide variety of platforms, the model training procedure tests a series of variables on a training set of true positive (TP) and false positive (FP) variants. For example, there are 29 and 16 variables evaluated for SNPs and INDELs for SOLiD, respectively (Additional file 1, Tables S1 and S2 and Figures S2 and S5). While variables that are significant for a specific platform may vary, we have established a semi-automated pipeline for re-training the regression models for different platforms. The Atlas2 Suite is written in a modularized manner that allows us to rebuild the regression models for new types of data and integrate it into the application in just a few days. This ability ensures that the Atlas2 Suite remains as an up-to-date and effective variant calling tool in the midst of rapidly evolving sequencing technologies.

### Statistical Model in Atlas2

In SOLiD Atlas-SNP2, we found five variables and two interactions to be most significant in regression model training: reference/variant reads ratio, mean variant base quality, mean neighboring base quality (NBQ), mean variant distance to the 3' end, strand direction standard, the interaction between NBQ and mean distance to 3' end, and the interaction between strand direction standard and mean distance to 3' end (Table 1). The reference/variant reads ratio acts as a simple measure of the direct evidence for and against the variant. As expected, sequencing and mapping errors generally have a much higher reference/variant reads ratio than true SNPs (Additional file 1, Figure S2a). The mean variant base quality takes the mean of each variant read's base quality at the SNP locus as reported by the sequencer. This provides a measure of the overall sequencing reliability of the variant bases. The NBQ is defined as the mean base quality score of the variant base and its 5 flanking base pairs (bp) on either side of the read. NBQ is a quantitative variation of the neighboring quality standard which has already been established as an effective metric in the Roche 454 version of Atlas-SNP2 and other variant calling pipelines [12]. The hypothesis behind this variable is that even bases with relatively high base qualities may be false positives if they are surrounded by bases of very low quality scores [13]; also reads with multiple sequencing errors are more likely to be mismapped. The mean variant distance to the 3' end is the mean distance from the variant site to the 3' end of the read in all variant reads. Sequencing quality generally diminishes towards the end of each read, so SNPs near the 3' end are often of lower quality (Additional file 1, Figure S2a). The strand direction standard is a Boolean variable which is set to 1 when there is at least one variant read in each strand direction. For true variants, the variant read strand direction is expected to follow a binary distribution with a roughly 50% probability for each direction; however, false variants are often found in only a single strand direction even when there is a large number of variant reads. In our training data we found that only 14% of the false SNPs had evidence in both strand directions, compared 94% for the true SNPs (Additional file 1, Figure S2a).

**Figure 2:**
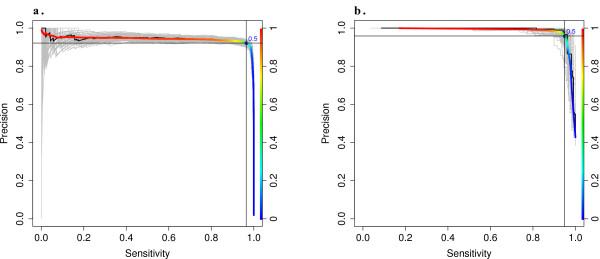
**Theoretical Performance of the Regression Models**. (a) The Atlas-SNP2 model is evaluated on a subset of the training data, which requires a minimum total depth of 10 base-pairs. (b) The Atlas-Indel2 model is evaluated on a subset of the training data that requires at least 2 variant reads (a default heuristic filter). To estimate the effectiveness of the regression models and test for overfitting, a series of cross-validation tests were performed by repeatedly sampling half of the training data to be used to train the model, and then evaluating the model on the remaining data. This process was repeated 100 times, with each result plotted as a gray line. The average of all these lines is plotted as a bold, color-coded line. The color indicates the *p *cutoff which returns the given performance at that point. The suite's default cutoff of 0.5 is marked. The actual model evaluated on the full set is plotted as a black line, but is mostly covered by the average line.

By performing a simple cross-validation procedure within the training data (see Methods), we were able to test the model for overfitting and provide an *in silico *estimate of its performance (Figure 2). The model was evaluated at all possible *p *cutoffs in terms of precision ($\frac{TP}{TP+FP}$) and sensitivity ($\frac{TP}{TP+FN}$). Results estimate the model is able to achieve a 97% recall rate and a precision of no less than 90% (limited sensitivity in the training data set caused some TP SNPs to be classified as FP, lowering the estimated precision). At the optimal cutoff of *p *≥ 0.5, the average performance of all cross validation iterations almost exactly matches that of the actual model evaluated on all training data, indicating that the model is not overfit.

Atlas-Indel2 uses a similar logistic regression model to separate true-positive and false-positive INDELs. During model training we discovered the most significant variables for INDEL calling to be the normalized variant square (NVS), the mean NBQ, the mean variation rate of the reads, and the read-end ratio (Table 1). The NVS is the number of variant reads squared and divided by the total read depth. In our initial testing the variant read depth was found to be extremely significant while the variant read ratio (variant read depth divided by total read depth) was not significant enough to be included in the model. This is partially due to the smaller INDEL training set which limited the number of variables we could include in the model without overfitting. We were concerned that using the variant read depth without any type of variant read ratio variable would introduce too much bias based on total coverage, which may vary considerably, especially from experiment to experiment. Our solution was to square the variant reads in the variant read ratio to give the NVS, which retains the significance of the variant read depth, but is normalized by the total read depth to remove any bias. Effectively, the NVS allows the model to consider both the variant read depth and the variant read ratio together in a single variable. The mean NBQ variable is defined the same as for the Atlas-SNP2 model. The variation rate is calculated by counting the number of mismatches and gaps in a read and dividing by the read length. While the reported mapping quality was not found to be at all significant (also it is too dependent on individual mapping software), the mean variation rate provides a simple indication of the mapping quality of the variant reads that was found to be highly significant. The read-end ratio variable is the number of reads where the INDEL is within 5 base-pairs of either read-end divided by the total number of variant reads. While visually inspecting FP INDELs, we noticed several were found primarily at the end of reads. This can be attributed to both the lower sequencing quality near the 3' end and the difficulty in mapping INDELs near read-ends. The read-end ratio variable was designed to identify these types of errors.

*In silico *cross-validation testing indicates that for INDELs that posses at least 2 variant reads (a default requirement of the heuristic filtering) the regression model is capable of calling INDELs with ~95% sensitivity and ~96% precision (Figure 2b). The regression model does not perform well in cases where only a single variant read is present. Our testing results indicate that the INDEL model is not overfit, with the average performance of all cross validation iterations closely matching the actual model's performance.

The INDEL logistic regression model was trained on a validated call set from human sample sequencing data (see Methods, and the Supplement (Additional file 1) for details). Although the variables tested during the model training included the variables used in the Atlas-SNP2 model, only the mean NBQ variable is included in both the SNP and INDEL models. The difference in which variables are most significant may be attributed to the different nature of error patterns around SNPs and INDELs. For example, while the SNP model uses the mean distance to the 3' end, the INDEL model uses the read end ratio variable. Both variables measure a similar metric, but while the distance to 3' variable is primarily related to detecting sequencing errors, the read end ratio is also useful in identifying mapping errors, which are the primary cause of false INDELs. As a result, the read end ratio is more significant than the distance to 3' in the INDEL model. An example of a more practical difference is that while the variant base quality is a clearly significant variable for SNPs, such a variable cannot be used for deletions which have no sequenced base or base quality.

The Atlas2 Suite also includes models for variant calling data from the Illumina platform. The Illumina SNP model is described in detail in our previous publication, where we have shown it to have high precision and sensitivity (90%, 95%, respectively) [10]. Our INDEL Illumina model is described in detail in the Supplement (Additional file 1), and is estimated to have high precision and sensitivity (93%, 86%, Additional file 1, Figure S4).

## Results

To evaluate the performance of the Atlas2 Suite, we processed 92 samples from the 1000 Genomes (1000G) Phase 1 Whole Exome Project http://www.1000genomes.org, which is an ongoing large scale population resequencing project aiming to provide the most comprehensive human variant call set [14]. These 92 samples were chosen because they are also included in the 1000G Exon Pilot Project [14,15]. For INDELs, we additionally analyzed 10 samples from the 1000G Whole Exome Project for comparison against other INDEL callers.

**Figure 3:**
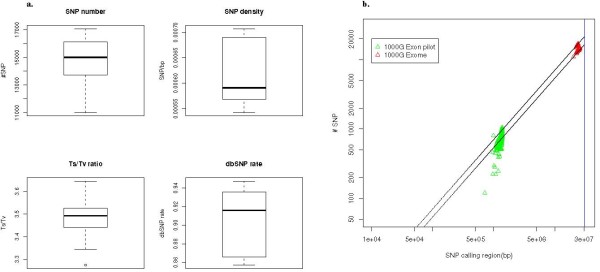
**SNP Call Metrics**. (a) SNP metrics of 92 1000 Genomes exome samples. The four figures are SNP number, Ts/Tv, dbSNP% and SNP density distributions respectively. SNPs were called and compared in the callable region with a variant read depth of at least 2. Previous studies have indicated that coding SNPs should have a Ts/Tv ratio of 3-4 [15]. (b) SNP density in the 1000 Genomes Exome Project vs. the Exon Pilot. The SNP density was calculated as the number of SNPs discovered in each sample normalized against their callable region. The maximum and minimum SNP density in the 1000 Genomes exome data are 0.70/Kbp and 0.54/Kbp respectively, which are presented as two slope lines in the figure.

In the 92 samples a total of 134,182 SNPs were discovered, with an average of 14,867 SNPs per sample (Figure 3a). Previous studies have established an expected transition/transversion (Ts/Tv) ratio of 3-4 for coding regions [15]. The Atlas-SNP2 call set has a Ts/Tv ratio of 3.49 and a dbSNP v129b re-discovery rate of 90.1%. We also checked the SNP density by normalizing the number of SNPs against each sample's callable region. The average SNP density of the 92 exomes was 0.63 SNP per 1,000 bp, which is consistent with previous results in the 1000 G Exon Pilot data (Figure 3b)

**Figure 4:**
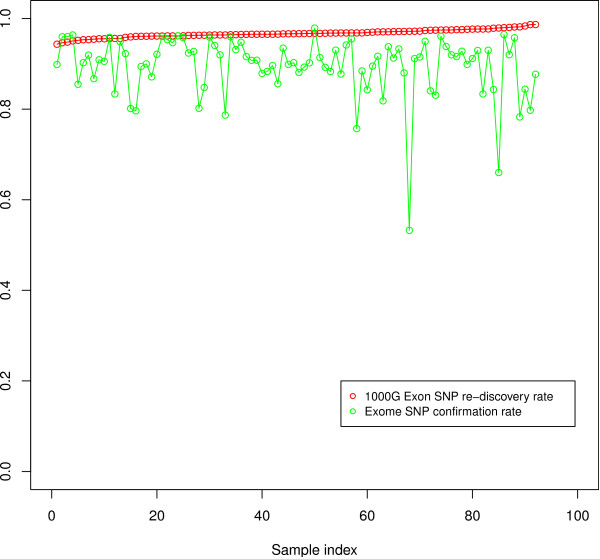
**Comparison of 92 1000 Genomes Exome samples to Exome Pilot Data**. We made SNP calls using the Atlas2 Suite on 92 samples from 1000 Genomes Phase 1 Exome project, and compared the result to the most recent release from the 1000 Genomes Exon Pilot project. The Atlas2 re-discovery rate was calculated for each sample (red). The average re-discovery rate is 96.7%. An average 89.5% of the SNPs called by Atlas2 were confirmed in the Exon Pilot project (green). The exome SNPs called by Atlas2 but not called in 1 KG Exon pilot is either due to Exon pilot's limited sensitivity or false discovery in Atlas2.

We compared the SNP calls of Atlas-SNP2 to the latest official release of 1000 G Exon pilot SNP calls (Aug 2010) on a sample-by-sample basis (Figure 4). Only SNP calls on the consensus high coverage genomic region of the two data sets were compared. On average, Atlas-SNP2 re-discovered 96.7% of the SNPs called in 1000 G Exon project on the consensus region. 89.5% of the SNPs discovered in 1000 G Exome data by Atlas-SNP2 are confirmed by the 1000 G Exon project data. With a Ts/Tv ratio of 3.21 and a dbSNP re-discovery rate of 71.1%, the remaining 10.5% of SNPs unique to the exome call set are likely to be true SNPs not discovered in the Exon project, which resulted from the stringent calling procedure employed by the Exon Pilot Project [15] as well as the evolving nature of the capture sequencing technology.

In the 92 samples we ran through the Atlas2 Suite pipeline, we called a total of 2,971 INDELs, with an average of 197 INDELs per sample (autosomal exons only). Frameshift INDELs in coding DNA nearly always render the resulting protein non-functional, and are expected to be significantly less common than in non-coding DNA. Previous studies have indicated that approximately 50% of coding INDELs cause frameshifts [16]. The samples analyzed by Atlas-Indel2 were found to have an average in-frame rate (ratio of INDELs that do not cause frameshift events) of 46.7%, indicating the call set may be of high quality.

For the purpose of comparison against other variant calling tools, 10 samples (5 European, 5 African) were processed by Atlas-Indel2, GATK [12], and SAMtools mPileup [8] (see Methods). Results were compared on the basis of total exome INDELs called and the percent of the INDELS that are in-frame (Table 2). The comparison shows that the Atlas-Indel2 call set has a significantly higher average in-frame rate of 47.52% compared to 10.39% for GATK and 25.82% for SAMtools mPileup (p < 3.9e-13 in a Student's t-test). In these 10 samples, Atlas-Indel2 called an average of 194 INDELs per sample, while GATK called 1,947 INDELs per sample and SAMtools called 1,560 INDELs per sample. 194 INDELs per sample is much closer to the number found in previous exome INDEL studies [14,17]. The low in-frame rate and large call set size for GATK and SAMtools indicate a much higher false-positive rate compared to Atlas-Indel2 (Additional file 1, Figure S9a and Additional file 2).

**Table 2:** Comparison to other INDEL Callers

	Atlas-Indel2	GATK Unified Genotyper	SAMtools mpileup
**Average INDELs/sample** **(Coding and Non-coding)**	23525	9648	26139

**Average Coding INDELs/sample**	194	1947	1560

**Average % 3(n) Coding INDELs/sample**	47.52	10.39	25.82

**# Coding INDELs**	816	12027	12305

**% 3(n) Coding INDELs**	38.11	7.78	23.84

**# Non-coding INDELs**	19607	3441	28135

**% 3(n) Non-coding INDELs**	14.06	9.79	17.19

Summary of INDELs called by Atlas-Indel2, GATK Unified Genotyper and SAMtools mPileup on 10 SOLiD samples (5 LWK, 5 CEU). The metrics compared are the average number of coding and non-coding INDELs per sample, the number of INDEL alleles merged across all 10 samples and the % 3(n) INDELs. The 3(n) INDELs refer to INDELs with a length of multiples of 3, which do not cause a frameshift mutation in the coding region. Previous studies have reported that coding regions tend to harbor less frameshift-causing INDELs. Coding refers to the consensus exome target regions of the genome as defined by the 1000 Genomes consortium. Non-coding refers to all the regions outside of the exome target regions. In the merged call sets, INDELs at the same site found in different samples are merged together in a population VCF file. Individual sample results are shown in Additional file 2.

Atlas-Indel2 is also specifically tuned for the Illumina platform in short INDEL calling and genotyping. The model is described in detail in the Supplement (Additional file 1, Table S3). As with the SOLiD data, we analyzed a small number of Illumina samples and compared the results of Atlas2 to a few other widely used INDEL callers including Dindel [18] and GATK [12]. The results show that all callers performed very similarly, calling between 221-241 average coding INDELs per sample with an average in-frame rate of 57-61% (Additional file 1, Table S5). 86% of the INDEL sites called by Atlas-Indel2 were also called by Dindel and 89% were also called by GATK (Additional file 1, Figure S9b).

### Computational Performance

**Figure 5:**
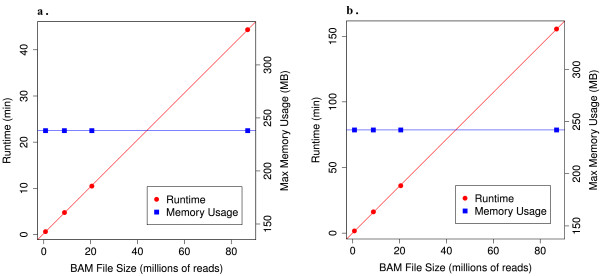
**Computational resources**. Both Atlas-SNP2 (a.) and Atlas-Indel2 (b.) were tested on a series of BAM files to evaluate run time and maximum memory usage. The algorithm for both applications is designed so that runtime increases linearly with the number of reads analyzed, while memory usage is approximately constant, based on the size of the reference genome.

The regular size of WECS BAMs is 10-20 gigabytes per BAM. Despite the enormous size of high coverage sequencing data, the Atlas2 Suite is engineered in a manner that allows it to process WECS data on a standard desktop computer or a small server in a reasonable amount of time. Sequencing reads are processed one at a time, with a minimal number stored in memory. The result is that the run-time increases linearly with the BAM file size and memory usage remains constant (Figure 5). Memory usage is dependant on the reference genome used; for example, using the human reference genome the maximum memory usage is about 250 MB. A whole exome 28 GB BAM file will take approximately 2 hours to be processed by Atlas-SNP2 and 5.5 hours by Atlas-Indel2, using one core of a 2.27 GHz Intel Xeon Processor. Using 64 CPU cores on a computational cluster we are able to process all 92 samples in ~4 hours for SNPs and ~11 hours for INDELs, demonstrating that the Atlas2 Suite is well suited for population scale analysis.

### Using Atlas2 in the Genboree Workbench for Integrative Genomic Analysis

In order to make the Atlas2 Suite tools accessible to a wide range of researchers we integrated the tools into the Genboree Workbench (http://www.genboree.org, registration required). Primarily aimed at collaborative genomics research, Genboree provides web-based services for groups of researchers to share, visualize, and analyze genomic annotations and raw data files. Using the graphical user interface at the Workbench, researchers can run Atlas2 Suite tools on their BAM and SAM files (Additional file 1, Figure S7a). The interface provides help information, validation of parameters, and adds the ability to upload the SNP and INDEL calls as annotation tracks. Upon submission, the configured job is added to the Genboree job queue and will execute on a modest compute cluster, after which the user is notified of job completion via email. The result files are also made available to the collaborating researchers via the Workbench, and are organized in a directory structure using the Study and Job names provided by the researcher (Additional file 1, Figure S7b).

By running the Atlas2 Suite tools via the Workbench, researchers benefit from integrative analysis. For example, if the SNP calls are uploaded as an annotation track, they can be viewed visually in the internal genome browser (Additional file 1, Figure S7b). Researchers can also configure Genboree to export their SNP tracks to the UCSC browser, and can use the tracks as inputs to other Workbench tools. Additionally, the Workbench provides us with a framework for adding new Altas2 Suite models and additional external tools. Because the Workbench is implemented on top of the Genboree HTTP REST API, researchers can automate this kind of analysis. For example, BAM file upload and launching an Atlas2 Suite tool can be done through the API, as can checking if result files are available and, if so, downloading them. Using the APIs, the researchers can also extend the analysis capabilities using their own software.

## Conclusions

As exome capture continues to become an effective method for high coverage gene sequencing and mutation discovery in personalized diagnosis, there is strong demand for a complementary set of data analysis tools specialized for the unique error models and other challenges inherent to exome capture. The Atlas2 Suite provides an effective and flexible solution to exome variant detection and genotyping, using statistical methods trained on exon capture data. We have applied our method to 92 whole exome human samples and demonstrated the suite is capable of producing sensitive and accurate variant calls in a reasonable amount of time.

Integration of the Atlas2 Suite into the Genboree Workbench provides the benefits of a visual interface, the easy sharing of access-controlled data, a hierarchy for organizing results of multiple analysis jobs, the potential for integrative analysis, and automation support. These additional resources provide a valuable online tool set to the wider research community.

The results of the Atlas2 Suite model development are beneficial not only for use in calling variants, but also for classifying variants and identifying error models for exon capture data that can be applied in a more general sense. For example, it was somewhat unexpected to discover that for SNPs the base quality score of the variant site is less significant than the base qualities of the surrounding bases in the mean NBQ; the number of variant reads was not even significant enough to be included in the final model (Additional file 1, Figure S2). We discovered that the most significant variable in SNP calling was the reference/variant read ratio. Almost all true SNPs had a reference/variant ratio less than 5, while most false SNPs had a ratio greater than 20. The most surprising discovery in the INDEL model training was that the mapping quality had almost no significance and could not be used to improve INDEL calls. We found, however, that the variation rate of the reads (penalizing both SNPs and INDEL equally) provided a better surrogate to capture the alignment quality in a region that harbors an INDEL.

While highly accurate SNP calling (sensitivity and precision > 95%) has become a very achievable goal using current methods, such consistent results remain a serious challenge for INDEL calling. In our comparison of the different SOLiD INDEL callers over 90% of the INDEL calls were unique to one of the callers (Additional file 1, Figure S9a). The overlap looks much better in Illumina comparison, but there are still 20% of the INDEL calls which were unique to one of the callers (Additional file 1, Figure S9b). The short read length and high error rate of NGS technology makes INDEL calling inherently challenging (especially for insertions). In order to gain significant improvement we will likely need to widen our approach to include other methods such as *de novo *or guided assembly.

We intend to continue maintenance and improvement of the Atlas2 Suite through a series of regular releases. Atlas2 will be updated as training data becomes available on new sequencing platforms.

## Methods

### Model training

Training the logistic regression models was performed in a semi-automated fashion. All training was performed using the R statistical environment. Each variable was applied to the training set and split by TP/TN status (Additional file1, Figures S2 and S5). Variable selection and model training was automated using the "glm" and "step" commands, and then fine tuned manually to remove variable redundancy. Additional variables were manually dropped with the guidance of the "drop1" command when variables did not show high significance (*p *> 0.05), or when the model appeared to be overfitted.

We checked for model overfitting using two methods. First, we performed a series of cross-validation tests on the entire model, in which we sampled 50% of the training data, retrained the model on this selection, and then evaluated the model on the remaining 50%. This was performed a minimum of 100 times and the mean of the results was compared to the original, theoretical model by plotting the model performance with a receiver operating characteristic (ROC) and precision/sensitivity curve (Figure 2). When the model is overfit there is high variance in the cross-validation tests and the mean performance differs significantly from the theoretical performance. The second method for detecting overfitting was a standard bootstrap procedure performed on the model using the R-command "boot" and "boot.ci" to determine the 95% confidence interval of each of the variable coefficients. An unreasonably wide confidence interval is expected when the model is overfit.

The training set was also used for tuning of the default *p *cutoff and other heuristic filters. An initial estimate of proper cutoffs and filters was established. Next, each individual cutoff or filter was evaluated at the full range of possible values with ROC and precision sensitivity curves. Cutoffs and filters were chosen that maximized the overall sensitivity and precision of the resulting call set.

#### SNP training data

The SNP logistic regression models were trained using a set of 10 overlapping samples from the 1000 Genomes Exon Pilot project (Exon) and the 1000 Genomes Exome project (Exome). We determined the overlapping callable region, which is defined as having high coverage (at least 10 reads) in both Exon and Exome data for each sample. The average region size is ~1.2M bp. The TP was made up of all the SNP candidate sites in Exome data having at least two variant reads and confirmed by Exon SNP calls. The FP comprised all other sites having at least two variant reads not confirmed by Exon SNP calls. In total, we have 6,516 TP and 347,154 FP in our training data set. Because the training data set is based on a continuous callable region, it represents the real TP/FP ratio in the sequencing data. An additional advantage of using the 1000 G Exon Pilot call set is that it provides a training set with high sensitivity among rare SNPs, [15] allowing us to create a final model that is not biased by allele frequency.

#### INDEL training data

The indel logistic regression models were created and trained on human exome capture SOLiD data that underwent PCR-Roche 454 validation. The TP in the training set were made up of the validated indels. The FP in the training set consisted of potential indels that were shown to be false-positives in the validation experiment plus a random selection of potential indels that were not in the initial indel call set. A very large number of FPs were selected to make the final training set 10% TPs and 90% FPs. This gives the final training set a more realistic TP/FP ratio to improve model training and provide more accurate estimations of the model's performance in real data. The training data was selected without regard to allele frequency and is all high-coverage exome capture data, which provides a realistic allele frequency distribution to ensure that the final model will not be biased against rare INDELs.

Once the INDEL training data was collected it was analyzed using the Atlas-Indel2 training scripts to calculate all the variables that can potentially be used in the logistic regression model (Additional file 1, Figure S5). The data was output in a tab-delimited format suitable to be imported into the R statistical package for model training.

The Atlas-Indel2 Illumina training data was created in the same manner as the SOLiD data, but the data was obtained from the validated INDEL calls of the Exon Pilot of the 1000 Genomes project [14,15].

### Evaluation and validation

#### Data Sources

All of the sequence data used for evaluation and validation of the Atlas2 Suite was obtained from the 1000 Genomes Project [14]. The samples were sequenced on the Applied Biosystems SOLiD 4 System and aligned using BFAST. The BAM files were processed using MarkDuplicates [19] and GATK local realigner [12].

#### Callable region in SNP discovery

The callable regions for the 92 SOLiD WECS samples were defined as the base positions with an effective read depth of at least 6. The effective read depth is the sum of reads harboring both a reference base and variant base, after excluding the reads failing the alignment/mapping quality cutoffs. For the SOLiD model, we required the effective reads to have at most three variant events (including SNPs and insertions and deletions) and the mapping quality score to be 255 that indicates a uniquely mapped read in BFAST alignments. All SNP calling, genotyping, and evaluation were performed within callable regions.

#### SNP calling and genotyping

SNPs were called using Atlas-SNP2 for SOLiD on the 1000 Genomes Project exome consensus target region with the default settings, which include: a SNP logistic regression model probability of *p *> = 0.5 and a variant read depth > = 2. The SNP sites were further genotyped using the adjusted variant ratio *t*, which is defined as the ratio of variant read depth to total read depth minus color corrected bases. SNPs with *t *> = 0.1 were genotyped as heterozygous SNPs and those with t > = 0.8 were genotyped as homozygous SNPs.

#### SNP evaluation

92 exome samples called by Atlas2 are also called in 1000 G Exon pilot. We performed a sample by sample specific comparison on the SNP calls of the consensus high coverage region of both Exome data and Exon pilot data. High coverage is defined as at least 10 × coverage in Exon pilot data and at least 6 × effective coverage and variant read depth ≥ 2 in exome data. Exon SNP re-discovery rate is defined as the ratio of SNPs called in the Exon pilot in the consensus high coverage region also called by Atlas2. Exome SNP confirmation rate is defined as the ratio of SNPs called by Atlas2 but not called in the Exon pilot.

#### INDEL calling

INDELs were called using Atlas-Indel2 in SOLiD data mode, with the default settings. Default settings include: a minimum *p *of 0.5, a minimum *p *of 0.88 for single base-pair deletions, a minimum of 2 variant reads, a minimum total depth of 2, and a minimum variant read ratio of 0.05. INDEL calls were filtered to only include INDELs located in the exome regions as defined in the 1000 Genomes consensus target regions. Individual sample VCF files were merged using the vcfPrinter tool.

#### INDEL evaluation

For comparison purposes, INDEL calls from Atlas-Indel2 were compared against calls made using Genome Analysis Toolkit (GATK) Unified Genotyper and SAMtools mpileup. Atlas-Indel2 was run with default settings and both GATK Unified Genotyper and SAMtools mpileup were run as described in their respective documentation pages (see Supplement in Additional file 1 for details). We took five samples from CEU and LWK populations and processed them with all three callers.

A total of 30 VCF files were created by running the 3 callers on 10 samples. Each VCF file was broken down into an on-target and off-target VCF file, based on the 1000 Genomes consensus target regions ftp://ftp-trace.ncbi.nih.gov/1000genomes/ftp/technical/working/20110228_consensus_exome_targets/20110225.exome.consensus.bed. A multi-sample VCF file was generated using vcfPrinter. Table 2 summarizes the results from the three INDEL callers. Only the autosomes are included. Supplementary table S4 (Additional file 1) reports the raw number of INDELs called by the different callers on each sample and the number of INDELs in on-and-off target regions.

## Availability and requirements

• **Project name**: Atlas2

• **Project homepage**: http://sourceforge.net/projects/atlas2/

• **Operating systems**: Unix based

• **Programming language**: C++, Ruby

• **Other Requirements**: SAMtools http://samtools.sourceforge.net/

• **License**: BSD

• **Any restrictions to use by non-academics: **none

• Atlas2 version 1.0 is included as Additional file 3

## Abbreviations

1000 G: The 1000 Genomes Project; BAM: binary sequence alignment/mapping format; bp: base pairs; CEU: Utah residents with Northern and Western European ancestry; Exon, FN: false negative; FP: false positive; GATK: Genome Analysis Toolkit; INDELs: insertion-deletions; LWK: Luhya in Webuye, Kenya; NBQ: mean neighboring base quality; NGS: next-generation sequencing; NVS: normalized variant square; *p*: probability; ROC: receiver operating characteristic; SNP: single nucleotide polymorphism; Ts/Tv: transition/transversion ratio; TN: true negative; TP: true positive; VCF: variant call format; WECS: whole exome capture sequencing.

## Authors' contributions

DC, JY and USE developed Atlas2, and carried out the analysis. ARJ, SP, CC and AM developed and maintain Genboree Workbench, and integrated the Atlas2 Suite with it. FY, RAG and AM conceived and directed the project. DC, JY, USE, ARJ, AM and FY wrote the manuscript. All authors have read and approved the final manuscript. The authors declare that they have no competing interests.

## Supplementary Material

Additional file 1 **The Supplementary Material for the paper**. Click here for file

Additional file 2 **Supplementary table S4, which is too large to include in the text**. Click here for file

Additional file 3 **The Atlas2 Suite version 1.0**. Click here for file
