# Supplementary material for: An integrative variant analysis suite for whole exome next-generation sequencing data
Source: BMC Bioinformatics. 2012 Jan 12;13:8. doi: 10.1186/1471-2105-13-8 (PMC3292476; doi:10.1186/1471-2105-13-8)

# Supplementary Materials

## Table of Contents

SNP OMNI Chip Comparison 2

SNP Model Details 2

Comparison of Atlas2 Illumina and SOLiD SNP Calls 3

INDEL Model Details 4

INDEL Illumina Model 4

Heuristic Filters and Genotyping for SOLiD/Illumina 5

Calling INDELS with GATK and mPileup 6

Atlas-Indel2 Comparison to Dindel and GATK on Illumina Data 7

Site overlap between the callers 8

Supplementary Tables 8

Supplementary Figure Legends 13

Supplementary Figures 16

## SNP OMNI Chip Comparison

We also made use of the Illumina OMNI2.5 BeadChip genotype data available from Phase 1 of the 1000 Genomes project. The OMNI2.5 chip genotypes about 2.4 million markers, including SNPs from the 1000 Genomes Pilot projects for all 92 samples. This provides a highly reliable set of true positive and true negative SNPs for call set validation, but it is limited by a lack of rare SNPs. Of the SNPs interrogated by the OMNI chip, we calculated an average confirmation rate of 99.8%, with values ranging from 99.2 to 100%.

## SNP Model Details

In contrast to the Atlas-SNP2 Illumina/454 models [10], in which the error probability of each variant bases is evaluated independently in a logistics regression model and then the posterior probability of each candidate site being a true SNP is calculated in a Bayesian framework, all variables are collected across the sites and the probability of a site being a true SNP is evaluated in a single step by the Atlas-SNP2 SOLiD logistic regression model.

The Atlas-SNP2 SOLiD model uses 5 variables and 2 interactions that are relevant to the sequence/platform context. The five variables are 1) the reference/variant reads ratio, 2) the mean neighboring base quality (NBQ) around the SNP, 3) the strand direction standard, 4) the mean distance to the 3’ end, and 5) the mean variant base quality (VBQ). The reference/variant reads ratio is simply the number of reference reads at the loci divided by the number of reads containing the variant of interest. The mean NBQ takes the mean base quality of the bases in a 10bp window around the SNP for each read, then averages all the values across reads. The strand direction standard is a Boolean variable that returns 1 if there is at least one variant read in each strand direction, otherwise 0. The mean distance to the 3’ end is calculated as the average of the distances of the variant base to the 3’ end of each variant read. The mean VBQ is the mean of the base qualities reported at the variant base.

The full regression model is shown below:

logit(*p*)= -19.18-0.4779×*ref_var_ratio*+0.2368×*mean_NBQ*+4.520×*strand_dir*+0.5436×*mean_dist3’*+0.08243×*mean_VBQ-*0.00796×*mean_NBQ*×*mean_dist_3’*-0.1122×*strand_dir*×*mean_dist_3’*

Before we settled on the linear model, we searched through the parameter space and tested a large number of potentially relevant variables. However they were not significant in our training results. These variables are described in Table S1.

## Comparison of Atlas2 Illumina and SOLiD SNP Calls

We compared the individual alternative allele count (AC) distribution of SNPs called in SOLiD and Illumina platforms (Figure S3). The AC are estimated in the whole population of 1000G whole exome project Phase1 (N=1128). Only samples of the same European population in different sequencing platforms (N=123 for Illumina, N=119 for SOLiD) are compared. The performance of Atlas2 is consistent for both SOLiD and Illumina data. For example, in both sequencing platforms, the number of singletons (AC=1) per sample on 1000G exome consensus target region is about 100-200 and the number of doubletons (AC=2) is about 50-75 etc.

## INDEL Model Details

The Atlas-Indel2 SOLiD model consists of four variables, the normalized variant square, the mean NBQ around the indel, the mean variation rate of the reads, and the ratio of reads where the indel is near the read end (Table 1). The normalized variant square is defined as the number of variant reads squared, divided by the total number of reads. The mean NBQ takes the mean base quality of the bases in a 10bp window around the indel for each read, then averages all these values for the final mean. The variation rate is calculated for each read by counting the number of mismatches and gaps in the read and averaging this ratio across all reads. The read-end ratio is the number of reads where the indel is within 5bp of either read end divided by the total number of variant reads. The full SOLiD indel regression model is shown below:

logit(*p*)=-4.51205+1.30088×*norm_var_square*+0.10516×*mean_NBQ*-72.16431×*mean_var_rate*-2.10047×*read_end_ratio*

We initially came up several variables and tested them using the training data, which indicated them as insignificant. These variables can be seen in Figure S5b and are described in Table S2.

### INDEL Illumina Model

Atlas-Indel2 also has the capability to process Illumina data. The Illumina model’s theoretical performance is shown in Figure S4. The Illumina model consists of four variables, the local sequence entropy, the strand direction, the normalized variant square, and the mean NBQ (Table S3). The local sequence entropy is obtained by calculating the entropy of the 20 base-pair subsequence around the INDEL, using the INDEL length as the window size. The strand direction is a binary variable that returns 1 if there is a variant read in each strand direction and otherwise returns 0. The full Illumina indel regression model is:

logit(*p*)=-20.5000+3.39065×*local_entropy*+3.02573×*strand_dir*+0.32695×*norm_var_square*+0.37184×*mean_NBQ*

While both the SOLiD and Illumina models include the normalized variant square and the mean NBQ variables, the Illumina model replaces the variation rate and read end ratio with the local entropy and the strand direction filter. This loss of the read-end ratio and variation rate variables is very reasonable considering the longer read length on the Illumina platform, which makes mapping errors somewhat less of a problem. The strand direction variable actually was fairly significant in the SOLiD data, but was displaced by more significant variables such as the variation rate. The local entropy variable appears to capture a class of sequencing errors specific to the Illumina platform, since it showed very little significance in the SOLiD data. In addition, the local entropy variable favors longer INDELs, which we found to be more reliable.

### Heuristic Filters and Genotyping for SOLiD/Illumina

While the logistic regression models are effective independent of any heuristics, we implemented some heuristic filters that users can enable and adjust to further improve INDEL call set accuracy and meet the needs of their experiments. Although these filters are similar to variables already considered in the regression models, we found we can make marginal gains in call set precision with the addition of a few lenient filters that capture the small number of edge-cases that the regression model misclassifies. The available filters include minimum total depth, minimum variant depth, minimum variant read ratio, a strand direction filter, and a near read-end filter. The strand direction filter requires that an indel has at least one supporting read in each strand direction. The near read-end filter removes INDELs in which more than the specified ratio of variant reads have the variant within 5 base-pairs of either read-end. The regression model’s *p* cutoff may be adjusted by users to enhance sensitivity or specificity. Atlas-Indel2 additionally allows users to specify a higher *p* cutoff for single base-pair (1bp) deletions to counter the high false-positive rate in this class of indels. For the SOLiD platform this cutoff is set to a default of 0.88.

Atlas-Indel2 also performs basic genotyping based on heuristic cutoffs. These genotypes should be considered low-confidence and preliminary to a more robust genotyping method. The basic genotyping performed by Atlas-Indel2 is based on the major variant depth coverage divided by the sum of the major variant depth coverage and the reference depth coverage: *var/(var+ref)*. When this ratio is greater than a user defined cutoff, the indel is called as a homozygous variant. If the ratio is less than or equal to the cutoff and above the minimum variant read ratio cutoff, the indel is called as a heterozygous variant.

## Calling INDELS with GATK and mPileup

Our INDEL calls made with Atlas-Indel2 were compared against calls made by the Genome Analysis Toolkit (GATK) Unified Genotyper (version 1.0.5974) and using the SAMTools (version 0.1.12a) mpileup variant calling pipeline.

GATK Unified Genotyper was run on individual BAMs with the -glm option set to indel so as to only output INDELs. All the other options were set to default.

SAMtools mpileup variant calling pipeline was also run in single sample mode and consisted of three steps: 1. the first step involved generating a raw binary variant call format file (bcf). SAMTools mpileup was run with -u, -g and -f flags: -u to output non-compressed binary variant calls, -g to generate bcf files and -f to pass a reference fasta file. The second step involved running bcftools view to convert the bcf file into a variant call format file. Bcftools view was run with -v, -c and -g flags set: -v to output only variant sites, -c for SNP calling and -g to output genotypes for variant sites. The output from bcftools view was filtered to remove the non INDEL variants. The third step was to filter raw INDEL calls made by mpileup using the vcfutils.pl script provided along with the SAMTools package. Vcfutils.pl script was run with the varFilter option with default settings.

## Atlas-Indel2 Comparison to Dindel and GATK on Illumina Data

We compared the Atlas-Indel2 calls on 3 Illumina sequenced samples from the 1000 Genomes project to INDEL calls made by Dindel and GATK on the same samples (Table S5). Since the number of coding INDEL alleles found by the three callers were very similar we looked for INDEL site overlap between Atlas-Indel2 and each of the callers. We found that 89% and 86% of the INDEL sites found by Atlas-Indel2 were also confirmed by GATK Unified Genotyper and Dindel respectively.

These samples were aligned using Burrows-Wheeler Aligner (BWA) and locally realigned using GATK local realigner [12]. GATK Unified Genotyper was run using default settings with the -glm option set to Indel to only output INDELs. Dindel was run as outlined in the user manual in section 5 outlining the basic steps for calling INDELs from a diploid sample (Table S5). Since the number of coding INDELs found by the three callers were very similar we looked for INDEL site overlap between Atlas-Indel2 and each of the callers.

**Site overlap between the callers**

We generated a Venn diagram showing the INDEL sites that overlap between the three callers on SOLiD and Illumina data (Figure S9). Call sets were compared on the site level i.e. exact coordinate position matches were called a hit without comparing the alternate alleles at that coordinate position. We calculated the percentages based on the union of the call sets.

# Supplementary Tables

## Table S1 – Description of tested SNP variables

| **Variable** | **Description** |
| --- | --- |
| Adjusted reference/variant read ratio | The effective base depth is used to calculate the reference/variant read ratio rather than the raw reads. |
| Total reads | The total read depth at the variant site. |
| Reference reads | The number of reference reads at the variant site. |
| Variant reads | The number of major variant reads at the variant site. |
| Variant read ratio | The variant reads divided by the total reads. |
| Adjusted reference reads | The effective base depth number of reference reads. |
| Adjusted total reads | The effective base depth at the variant site. |
| Adjusted variant read ratio | The effective base depth is used to calculated the variant read ratio rather than the raw reads. |
| Mean reference quality score (QS) | The mean base quality score of the reference reads at the variant site. |
| Strand score | This variable is 0 if there is at least one variant read in each strand direction; otherwise it is equal to the number of variant reads (higher is worse). |
| Mean reference color corrections (CM) | The mean number of color corrections in the reference reads. |
| Mean variant color corrections (CM) | The mean number of color corrections in the variant reads. |
| Mean total color corrections (CM) | The mean number of color corrections in all reads covering the variant site. |
| Mean reference position | The mean distance of the variant site from the left-aligned read end in all reference reads. |
| Mean variant position | The mean distance of the variant site from the left-aligned read end in all variant reads. |
| Mean reference distance to the 3’ end | The mean distance of the variant site to the 3’ end of the read in all reference reads. |
| Total color corrections (CM) | The total number of color corrections in all reads covering the variant site. |
| Normalized variant square | This is calculated by squaring the number of variant reads and dividing by the number of total reads. |
| Adjusted normalized variant square | The normalized variant square calculated using effective base depth rather than raw base depth. |
| Mean reference distance to center | The mean distance of the variant site to the center of the read in all reference reads. |
| Mean variant distance to center | The mean distance of the variant site to the center of the read in all variant reads. |
| Mean reference neighboring base quality (5bp window) | The mean NBQ in all reference reads using a 5 base-pair window. |
| Mean reference neighboring base quality (10bp window) | The mean NBQ in all reference reads using a 10 base-pair window. |
| Mean variant neighboring base quality (10bp window) | The mean NBQ in all variant reads using a 10 base-pair window. |

This table lists and describes the variables that were tested on the SOLiD SNP data during model training but were not included in the final model due to lack of significance, bias or other problems.

## Table S2 – Description of tested INDEL variables

| **Variable** | **Description** |
| --- | --- |
| Mean mapping quality | The mean of the mapping qualities of all variant reads |
| Length | The length of the INDEL. |
| Frameshift | A Boolean variable that returns 1 if the INDEL would cause a frameshift event (length mod 3 ≠ 0), otherwise returns 0. |
| Mean local entropy | Calculates the mean local entropy of all variant reads. The local entropy is evaluated by calculating the sequence entropy of the subsequence going from 10 base-pairs before the INDEL site to 10 base-pairs after the end of the INDEL. The indel length is used as the window size in the entropy function. |
| Variant reads | The number of major variant reads. |
| Variant read ratio | The number of variant reads divided by the total read depth. |
| Strand direction | A Boolean variable that returns 1 if there is at least one variant read in each strand direction, otherwise returns 0. |
| Strand score | This variable is 0 if there is at least one variant read in each strand direction; otherwise it is equal to the number of variant reads (higher is worse). |
| Lenient strand filter | A more lenient strand direction variable that only returns 0 if there are at least 5 variant reads and there is not a read in each strand direction. Otherwise returns 1. |
| Near read-end | A Boolean variable that returns 1 if all the variant site is within 5 base-pairs of the end of the read in all variant reads. Otherwise returns 0. |
| Near read-end plus strand direction | A Boolean variable that combines the near read-end and strand direction variables. It can be defined as *read-end and not strand direction*. |
| Mean color corrections | The mean number of color corrections on the variant reads. |

This table lists and describes the variables that were tested on the SOLiD INDEL data during model training but were not included in the final model due to lack of significance, bias or other problems.

## Table S3 – The Atlas-Indel2 Illumina Model

| **Variant Type** | **Description** | **Type** | **z-value** | **p-value** |
| --- | --- | --- | --- | --- |
| Indel | Normalized Variant Square (NVS) | Numeric | 4.054 | 5.04E-05 |
|  | Local sequence entropy | Numeric | 6.724 | 1.77E-11 |
|  | Strand direction | Boolean | 5.255 | 1.48E-07 |
|  | Mean neighbor base quality (NBQ) | Numeric | 3.752 | 1.36E-02 |

Variables in the Atlas-Indel2 Illumina logistic regression model. Variables included in the final regression model are shown along with the variable’s Wald z-statistic and p-value. The z-values and p-values indicate the significance of the variables in the model, with the most significant variables having a z-value furthest from zero.

## Table S4 – 10 Samples INDEL Comparison

[Included separately as Additional file 2]

The results of the INDEL calls for each individual sample are shown for the three INDEL callers. INDEL calls are compared both on-target and off-target by INDEL number, in-frame INDEL number, and percent of in-frame INDELs. The merged INDEL call sets (with INDELs at the same site merged across samples) are shown in bold. As expected the percent in-frame indels is lower in the merged call sets due to the higher frequency of in-frame INDELs compared to frameshift INDELs (see Figure S6).

## Table S5 – Comparison to INDEL Callers on Illumina Data

|  |  | **Atlas-Indel2** | **GATK Unified  Genotyper** | **Dindel** |
| --- | --- | --- | --- | --- |
| **Sample** | **Average INDELs/sample**  **(Coding and Non-coding)** | 16876 | 16680 | 45021 |
|  | **Average Coding INDELs/sample** | 241 | 225 | 222 |
|  | **Average % 3(n)**  **Coding INDELs/sample** | 56.56 | 59.53 | 61.27 |
| **Merged**  **Coding** | **# INDELs** | 454 | 416 | 408 |
|  | **% 3(n) INDELs** | 54.63 | 58.17 | 60.29 |
| **Merged**  **Non-coding** | **# INDELs** | 34065 | 31447 | 106155 |
|  | **% 3(n) INDELs** | 18.32 | 12.07 | 14.82 |

Summary of INDELs called by Atlas-Indel2, GATK Unified Genotyper and Dindel on 3 Illumina samples. The metrics compared are the average number of coding and non-coding INDELs per sample, the number of INDEL alleles merged across all 10 samples, and the % 3(n) INDELs. The 3(n) INDELs refer to INDELs with a length of multiples of 3, which do not cause a frameshift mutation in the coding region. Previous studies have reported that coding regions tend to harbor less frameshift-causing INDELs. Coding refers to the consensus exome target regions of the genome as defined by the 1000 Genomes consortium. Non-coding refers to all the regions outside of the target regions. Only the autosomes are included. In the merged call sets, INDELs at the same site found in different samples are merged together in a population VCF file.

# Supplementary Figure Legends:

## Figure S1 – MAF of Atlas2 Calls

The minor allele frequencies of both SNPs and INDELs in the 92 samples as called by the Atlas2 Suite are shown. As expected, a higher proportion of the INDELs are very rare due to the higher negative selection against INDEL variants.

## Figure S2 – Atlas-SNP2 Variable Distributions

The series of variables that were tested during training of the SOLiD Atlas-SNP2 model. Each variable is split up by true positive (TP) and true negative (TN) SNP in the training data. The final model was obtained by iteratively running the R package’s “step” command to evaluate different combinations of variables and removing redundant or problematic variables to produce the optimal model. (a)The 5 variables included in the final model. (b)The remaining variables that were tested but not included in the final model.

## Figure S3 – Individual allele frequency spectrum by sequencing platform.

The AC (alternative allele count) is estimated from the whole population of the 1000G Whole Exome Project Phase1 (N=1128). The box plots indicate the numbers of SNPs of all samples in the specified AC bin.

## Figure S4 – Theoretical performance of Atlas-Indel2 for Illumina data

The Illumina INDEL model was evaluated on the training data. This evaluation includes the default heuristic filters used for Illumina data. The color code indicates the *p* cut-off.

## Figure S5 – Atlas-Indel2 Variable Distributions

The series of variables that were tested during training of the SOLiD Atlas-Indel2 model. Each variable is split up by true positive (TP) and true negative (TN) SNP in the training data. The final model was obtained by iteratively running the R package’s “step” command to evaluate different combinations of variables and removing redundant or problematic variables to produce the optimal model. **A.** Variables included in the final INDEL model. **B.** Variables tested, but not included in the final model.

## Figure S6 – INDEL Alternative Allele Count

The alternative allele count of INDELs in the 92 samples is shown split by frameshift status. As expected, in-frame INDELs are significantly more common than frameshift INDELs. This was established using a Student’s t-test to compare the in-frame and frameshift INDEL alternative allele counts (p-value shown). The final bin of the histogram includes all indels with an allele count over 25.

## Figure S7 – Incorporation of Atlas2 Suite tools into the Genboree Workbench.

(a) Users select the BAM or SAM files to process and the database which will hold the output, and then pick the Atlas2 Suite tool they wish to run (a, left). The tool run is configured via a settings dialog and submitted to the Genboree job queue (a, right). (b) When the tool run completes, the researcher is notified by email and can then download the output files (b, left), view the SNP calls in the genome browser (b, right), or even use the annotation track as input to other Workbench tools.

## Figure S8 – Atlas2 work flow diagram in Genboree Workbench

To use Atlas2 tools in the Genboree Workbench, researchers typically first transfer a BAM or SAM file to Genboree; the SAM file can be in a compressed format. That BAM or SAM is then used as input to one of the Atlas2 SNP-calling tools. If the SNP calls were uploaded as annotation tracks, then collaborators can visualize the results in the Genboree browser or even configure Genboree to export their SNPs to the UCSC browser. The raw SNP call results are made available to the research group in several file formats, including VCF, and can be downloaded for examination and further analysis. Collaborators can easily re-run the genotyping using alternative parameters or even by providing their own raw result file.

## Figure S9 – Overlap between different INDEL callers

For all INDEL callers used as a comparison against Atlas-Indel2 we calculated the number of overlapping sites between their call sets and the Atlas2 call sets. This was performed for both SOLiD (a) and Illumina (b) data. The overlap is displayed as a Venn diagram with the percent of the union calculated for each section.

# Supplementary Figures

## Figure S1

## Figure S2

**
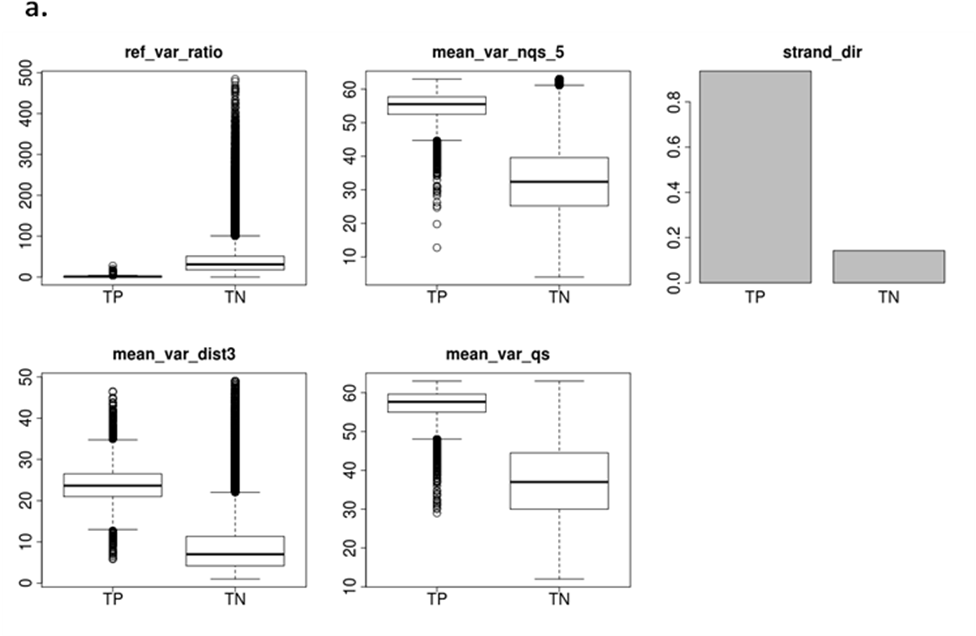
**

**
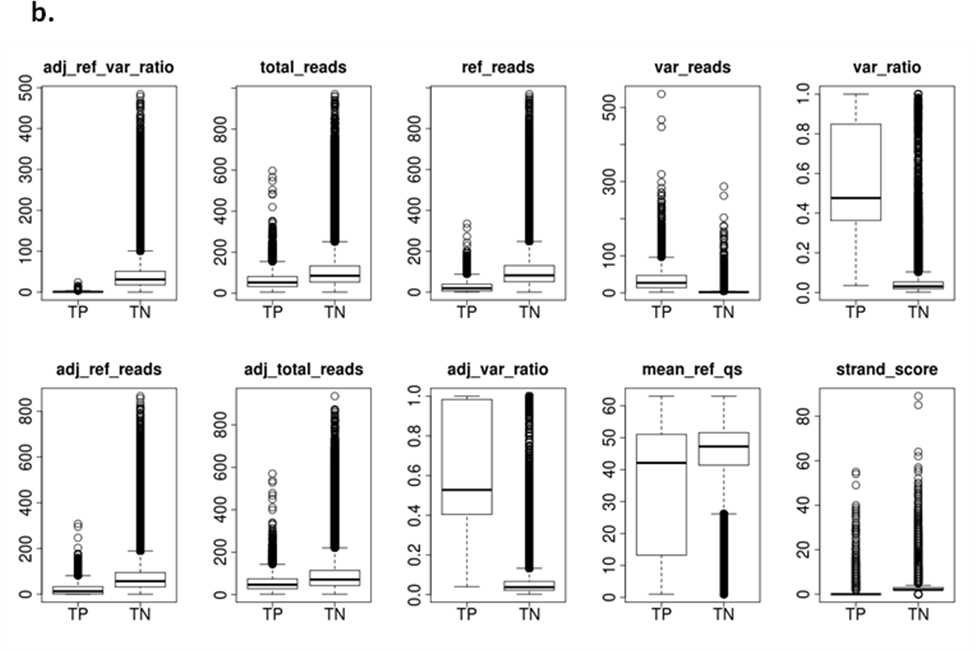

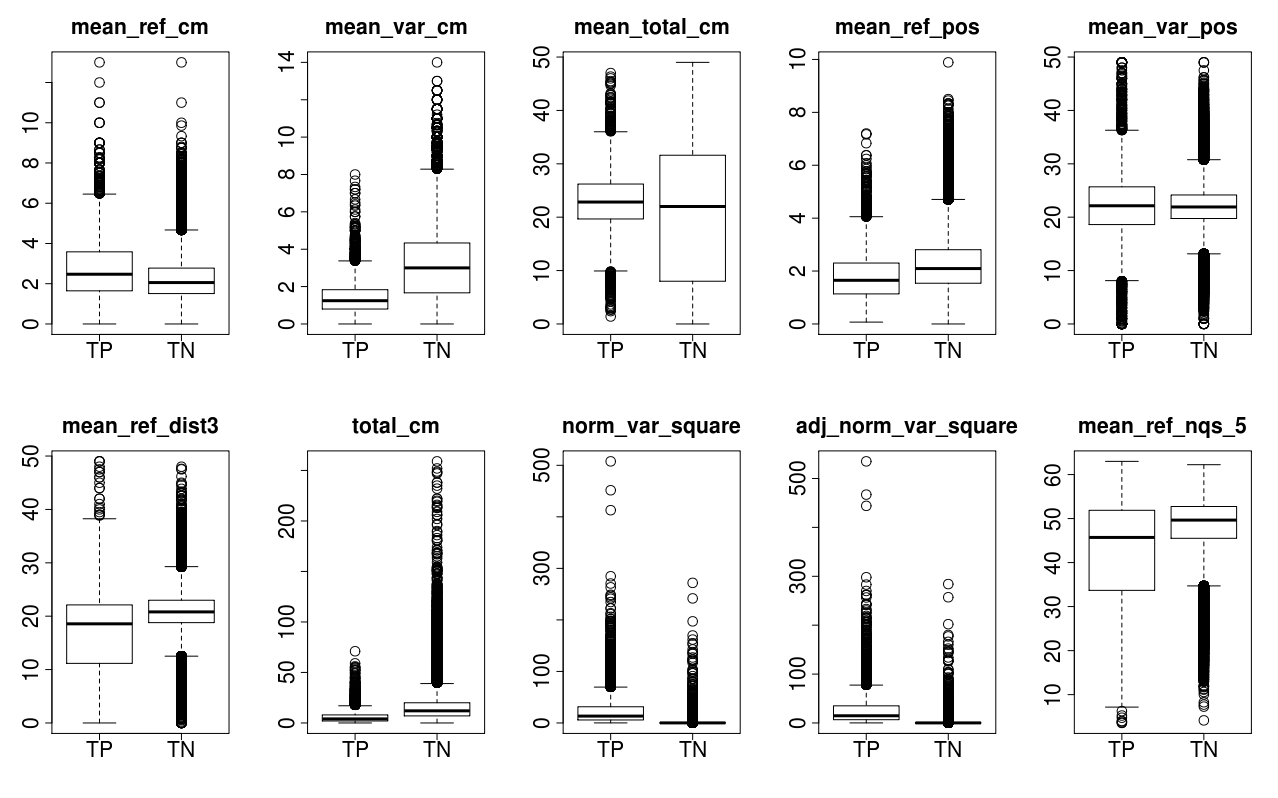
** **
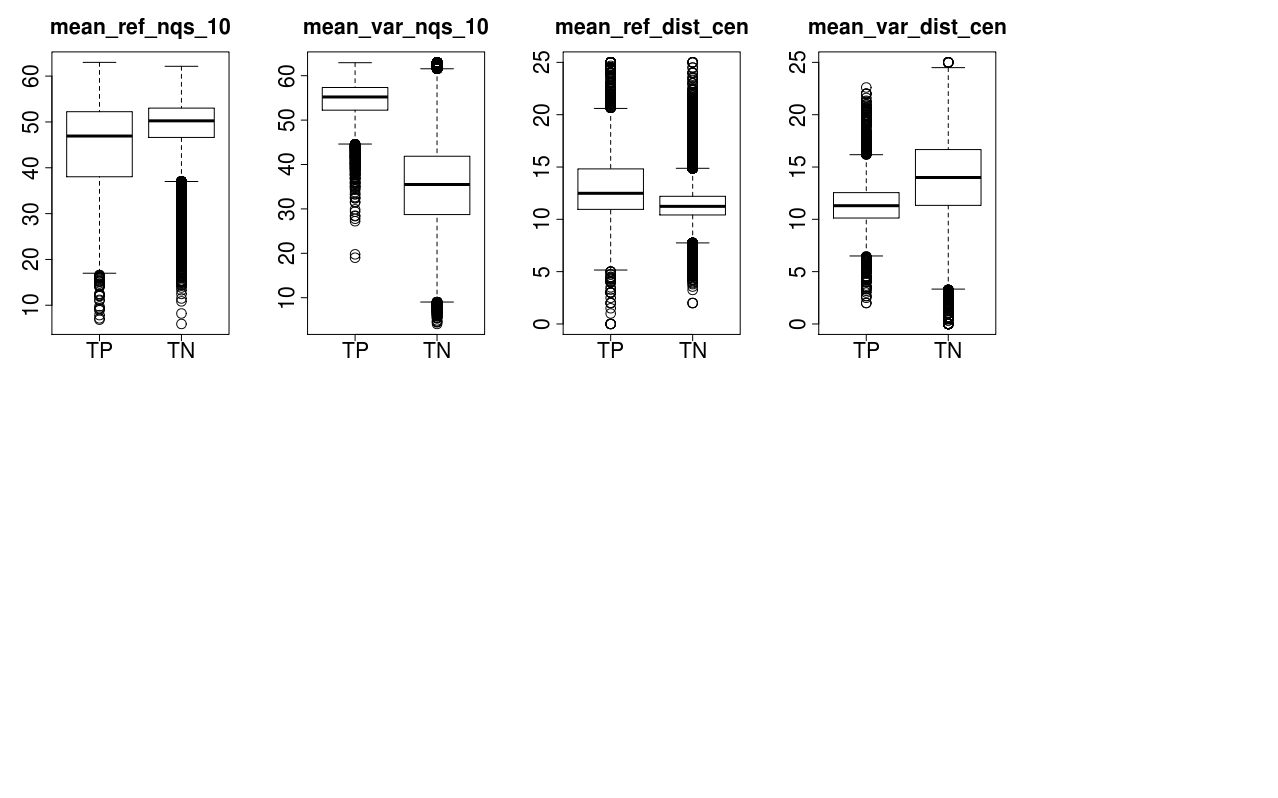
**

## Figure S3

**
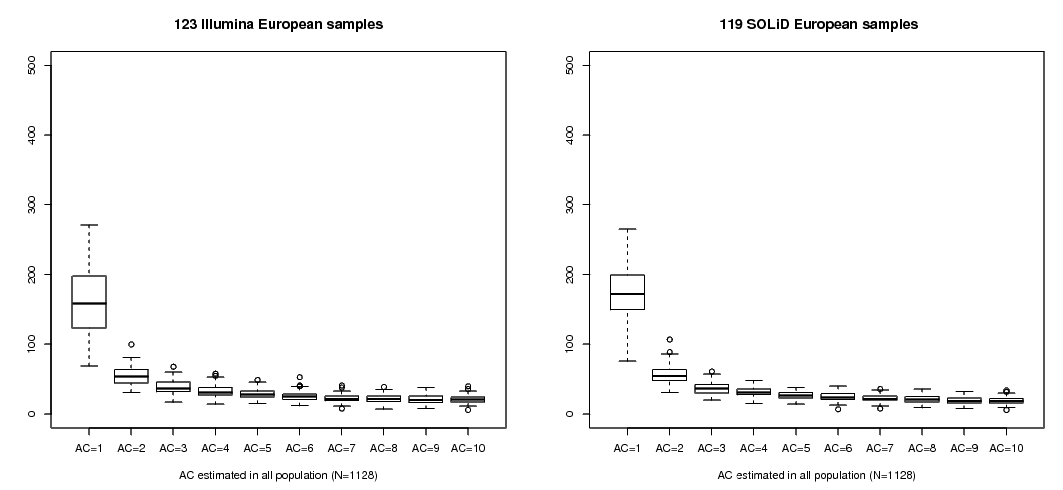
**

## Figure S4

## Figure S5

### a.

### b.

## Figure S6

## Figure S7

**
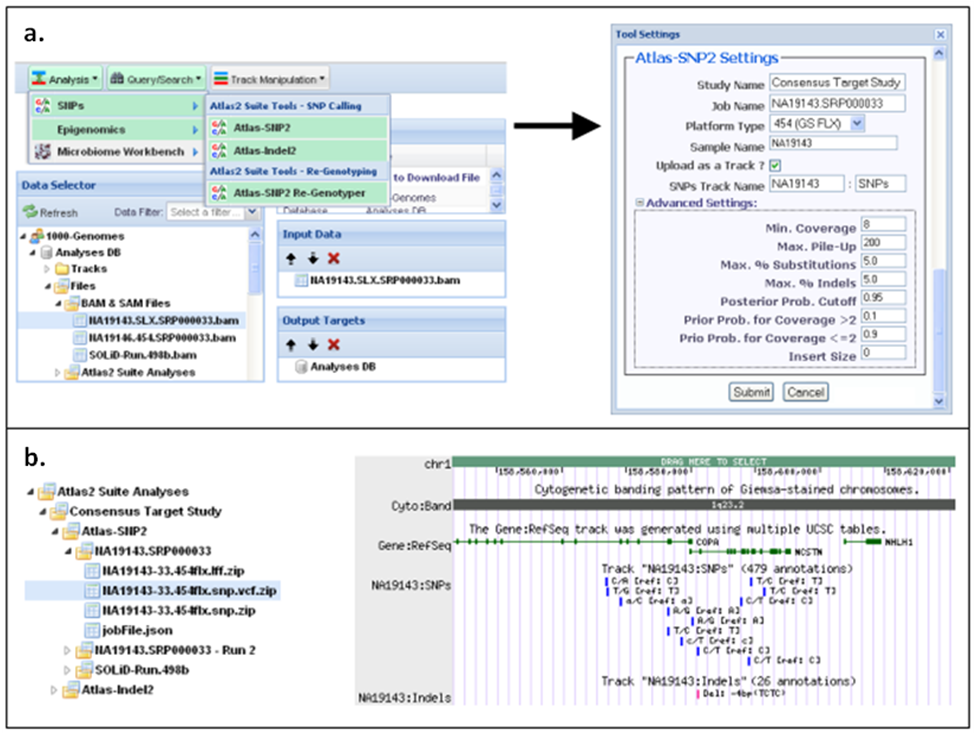
**

## Figure S8

**BAM**

**or**

**SAM File**

**Atlas-Indel2**

**Atlas-SNP2**

**Re-Genotyping**

**Examine:**

**Visualize**

**(genome browser)**

**Raw Result Files**

**(.vcf, .snp)**

## Figure S9

### a.


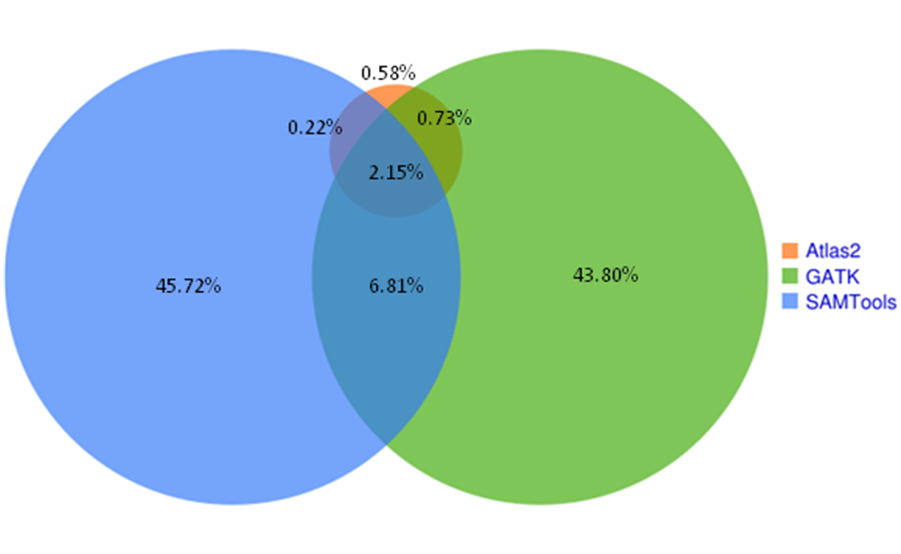


### b.


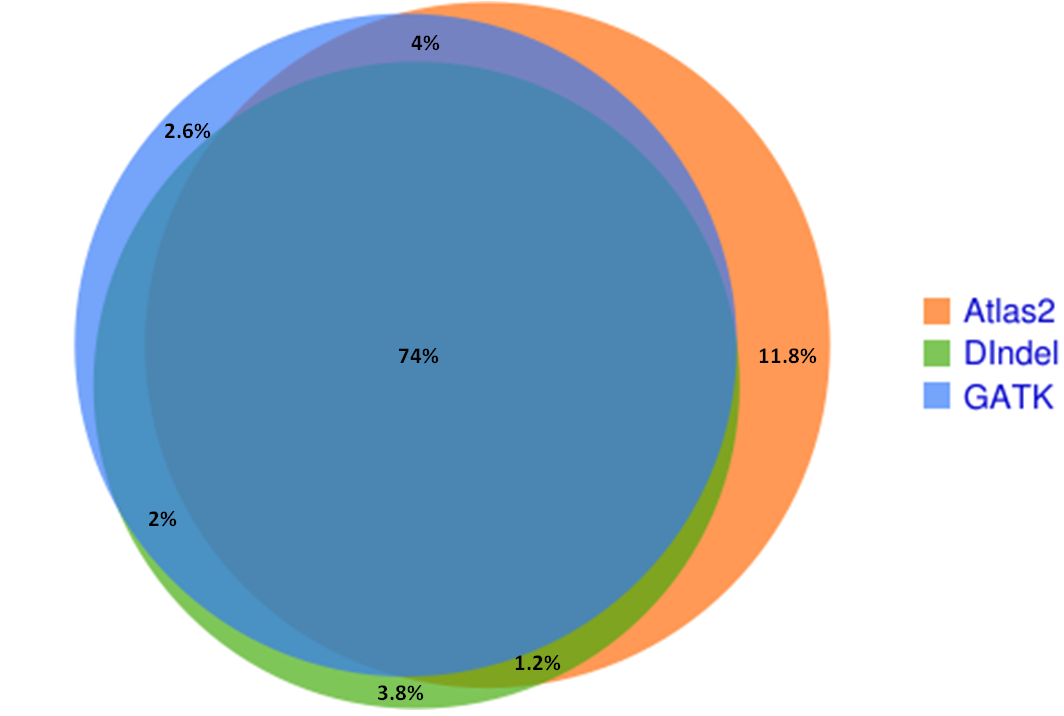

Supplement: Additional file 1 — The Supplementary Material for the paper. [file 1471-2105-13-8-S1.DOC]
