# Supplementary material for: An integrative variant analysis suite for whole exome next-generation sequencing data
Source: BMC Bioinformatics. 2012 Jan 12;13:8. doi: 10.1186/1471-2105-13-8 (PMC3292476; doi:10.1186/1471-2105-13-8)
Supplement: Additional file 3 — The Atlas2 Suite version 1.0. [file 1471-2105-13-8-S3.BZ2 › Atlas2_v1.0/Atlas2 documentation.pdf]

Atlas2 integrated SNP/INDEL calling pipeline

V 1.0 Aug 29, 2011

Contact: Danny Challis (challis@bcm.edu), Jin Yu (jy2@bcm.edu),

Uday Evani (Evani@bcm.edu) and Fuli Yu (fyu@bcm.edu)

Human Genome Sequencing Center (HGSC) at Baylor College of Medicine (BCM)

Houston, TX, USA

Table of Contents:

1. Introduction 3
2. System Requirements and Installation 3
3. Preprocessing 4
4. Usage 4
   4.1. Atlas-SNP2 4
   4.2. Atlas-Indel2 6
5. Output Format 8
6. References to Atlas2 9
7. References 9
8. Change Log 10
   8.1. Atlas-SNP2 10
   8.2. Atlas-Indel2 11
9. License 13
10. Appendix 13

1. Introduction

Atlas2 is a suite of variant analysis tools specializing in the separation of true SNPs and insertions and deletions (indels) from sequencing and mapping errors in Whole Exome Capture Sequecing (WECS) data. SNPs may be called using the Atlas-SNP2 application and indels may be called using the Atlas-Indel2 application. The suite implements logistic regression models trained on validated WECS data to identify the true variants. There is a separate regression model for each sequencing platform. The suite currently supports the SOLiD, Illumina, and Roche 454 (SNPs only) platforms. Future version of Atlas2 will include additional models for new sequencing platforms.

The Atlas2 suite takes a Binary sequence Alignment/Mapping (BAM) file (see http://samtools.sourceforge.net/SAM1.pdf) and a FASTA reference genome as input and produces variant calls in Variant Call Format (VCF) (see http://www.1000genomes.org/wiki/Analysis/vcf4.0). In addition to variant calls, the application collects coverage information and uses simple heuristic cutoffs to estimate the likely genotype of each variant site.

2. System Requirements and Installation

- Unix-like operation system
- Ruby 1.9.1+: http://www.ruby-lang.org/en/downloads/
- SAMtools must be installed and runnable by invoking the "samtools" command
    - SAMtools may be obtained at http://samtools.sourceforge.net/
- If you do not have a 64-bit Linux system, a C++ compiler and Make must be installed

SOLiD-SNP-caller is coded in C++ and must be compiled to run. For 64-bit Linux system this has already been done. If you have a different system take the following steps before running SOLiD-SNP-caller for the first time:

- Navigate to the SOLiD-SNP-caller directory in a terminal
- Run: `make clean`
- Run: `make`
- Compiling may take several minutes

3. Preprocessing

Although Atlas2 will function on any sorted BAM file, there are number of preprocessing steps that we recommend for the highest quality results.

- Mark or remove PCR duplicates using PicardMarkDuplicates (http://picard.sourceforge.net/index.shtml) or a similar tool.
- Base quality recalibration (not recommended for SOLiD data) using GATK (http://www.broadinstitute.org/gsa/wiki/index.php/The_Genome_Analysis_Toolkit) or a similar tool.
- Locally realign around likely indels and using GATK or SMRA (http://sourceforge.net/projects/srma/).

4. Usage

4.1 Atlas-SNP2

Atlas-SNP2 is designed to evaluate and distinguish true SNPs from sequencing and mapping errors in whole-exome capture sequencing (WECS) data.

a) For 454/Illumina data

```
ruby Atlas-SNP2.rb Atlas-SNP2.rb -i [in.sorted.bam] -r [reference.fa] -o
[output file] [choosing platform] [Setting up VCF output] [choosing
platform] [Setting up VCF output]
```

Atlas-SNP2 is coded in Ruby and basic usage can be viewed by running the program without any arguments.

-i	FILE	BAM format alignment file (Required to be sorted by start position)
-r	FILE	FASTA format reference sequence file (Required)
-o	STR	name of output result file (Required)
-t	STR	Only call SNPs on given target region (Optional, please refer "samtools view" for the target region format)

Choosing Platform: (Default is 454 FLX)
-s Illumina
-x 454 Titanium

Setting up VCF output
-v Output genotypes in VCF format
-n Sample name used in VCF file (Required when choosing VCF output)

-c Posterior probability cutoff (Default is 0.95)
-y Minimal Coverage required for high confidence SNP calls (Default is 8)

Setting up prior probability:
-e FLT Prior(error|c) when variant coverage number is above 2 for 454 and Illumina data (Default is 0.1)
-l FLT Prior(error|c) when variant coverage number is 1 or 2 for 454 data (Default is 0.9)

Setting up filters:
-m FLT maximum percentage of substitution bases allowed in the alignment (Default is 5.0)
-g FLT maximum percentage of insertion and deletion bases allowed in the alignment (Default is 5.0)
-f INT maximum number of alignments allowed to be piled up on a site (Default is 1024)
-p INT insertion size for pair-end re-sequencing data (Default is 0)

Example:

```
# Call SNPs of one Illumina BAM only on chr1 and output the SNP results in
VCF file ~/NA12275.chr1.snp.vcf

> ruby Atlas-SNP2.rb -i NA12275.bam -r ~/refs/human_g1k_v37.fasta -o
NA12275.chr1.snp -s -t chr1 -v -n NA12275
```

b) For SOLiD data

```
SOLiD-SNP-caller <in.bam> <ref.fa> [.bed region] > [output.vcf]
```

Basic usage can be viewed by running the program without any argument.

<in.bam> FILE BAM format alignment file (Required to be sorted by start position)
<ref.fa> FILE FASTA format reference sequence file (Required)
[.bed] FILE Only call SNP on given regions defined in bed format [optional]

Example:

```
# Call SNP of one SOLiD BAM only on coding regions and output the SNP
results in VCF file NA20532.ontarget.vcf

> SOLiD-SNP-caller NA20532.bam ~/refs/human_g1k_v37.fasta ccds.bed >
NA20532.ontarget.vcf
```

4.2 Atlas-Indel2

Atlas-Indel2 is designed to evaluate and distinguish true insertions and deletions (indels) from sequencing and mapping errors in whole-exome capture sequencing (WECS) data.

```
ruby Atlas-Indel2.rb -b [input_bam] -r [reference] -o [outfile] [-S/-I]
```

Basic usage information may be viewed at any time by running Atlas-Indel2 without any arguments.

Mandatory arguments:

-b, --bam=FILE

The input BAM file. It must be sorted. It does not need to be indexed. A read mask of 1796 is used on the bitwise flag.

-r, --reference=FILE

The reference sequence to be used in FASTA format. This must be the same version used in mapping the sequence. It does not need to be indexed.

-o, --outfile=FILENAME

The name of the output VCF file. Output is a simple VCFv4 file with a single sample. These files can be merged into a more complete VCF file using the vcfPrinter (included). If the file already exists, it will be overwritten. NOTE: For use with vcfPrinter, you should name your vcf file the same as your BAM file, simply replacing ".bam" with ".vcf".

–I or -S

You must include one of these flags to specify either the Illumina or SOLiD regression model to be used.

Optional arguments:

Note: Different platform modes have different defaults.

-s, --sample=STRING

The name of the sample to be listed in the output VCF file. If not specified the sample name is harvested from the input BAM file name, taking the first group of characters before a . (dot) is found. For example, with the filename "NA12275.chrom1.bam" the sample name would be "NA12275".

-p, --p-cutoff=FLOAT

Defaults: Illumina:0.5, Solid:0.5

The indel probability (p) cutoff value for the logistic regression model. Indels with a p less than this cutoff will not be called. Increasing this cutoff will increase specificity, but will lower sensitivity. If you adjust this cutoff, you should usually also adjust the p-1bp-cutoff (see below).

-P --p-1bp-cutoff
Defaults: Illumina:0.5, Solid:0.88
The indel probability (p) cutoff value for 1bp deletions. This may be set to a stricter standard than the normal p-cutoff to increase callset specificity. This is very useful for SOLiD data, but should not be generally needed for Illumina data.

-B --bed=FILE
Here you may specify a bed file which contians the region you wish to limit your indel calling to. Only reads inside the region will be process, which can significantly shorten the runtime.

-O --orig-base-qual
This is the default for SOLiD, it is not recommended for Illumina data. This option has the algorithm use the original base qualities as specified in the OQ tag if included in the BAM file. If the BAM file does not include OQ tags, the normal base quality is used.

-N --norm-base-qual
This is the default for Illumina, it is not recommended for SOLiD data. This option specifies the algorithm should use the normal base qualities, as specified in the QUAL column of the BAM file.

Heuristic Cutoffs:
Most of these variables have already been considered by the regression model, so you shouldn't usually need to alter them. However you are free to change them to meet your specific project requirements.

-t, --min-total-depth=INT
Defaults: Illumina:2, SOLiD:2
The minimum total depth coverage required at an indel site. Indels at a site with less depth coverage will not be called. Increasing this value will increase specificity, but lower sensitivity.
Suggested range: 2-12

-m, --min-var-reads=INT
Defaults: Illumina:2, SOLiD:2
The minimum number of variant reads required for an indel to be called. Increasing this number may increase specificity but will lower sensitivity.
Suggested range: 1-5

-v, --min-var-ratio=FLOAT
Defaults: Illumina:0.06, SOLiD:0.05

The variant-reads/total-reads cutoff. Indels with a ratio less than the specified value will not be called. Increasing this value may increase specificity, but will lower sensitivity.
Suggested range: 0-0.1

-f, --strand-dir-filter
Default: Illumina:disabled, SOLiD:disabled
When included, requires indels to have at least one variant read in each strand direction. This filter is effective at increasing the specificity, but also carries a heavy sensitivity cost.

-n, --near-read_end_ratio=FLOAT
Default: Illumina:0.8, SOLiD:1.0 (disabled)
The read end ratio is defined as the number of variant reads where the variant is within 5bp of a read end divided by the total variant read depth. If this ratio is greater than the specified value, the indel is filtered.
Suggested range: 0.7-1.0

-h, --homo-var-cutoff
Default: Illumina:0.6, Solid:0.5
The homozygous variant cutoff. This cutoff is used in the preliminary genotyping performed by Atlas-Indel2. If the variant reads divided by the variant reads + the reference reads is greater than this cutoff it will be marked as a homozygote, otherwise it will be marked as a heterozygote.

Examples:

```
> ruby Atlas-Indel2.rb -b NA12275.chrom1.bam -r
~/refs/human_g1k_v37.fasta -o ~/NA12275.chrom1.vcf -p 0.55 -I

> ruby Atlas-Indel2.rb -b seq1.10.2010.chrom1.bam -r
~/refs/human_g1k_v37.fasta -o ~/NA12275.chrom1.vcf -t 10 -m 5 -s NA12275
-B target_region.bed -S
```

5. Output Format

The output of Atlas2 is a simple VCF file. The sample column includes the estimated genotype (GT), the number of major variant reads (VR), the number of reference reads (RR) and the total read depth (DP) at that site. There are also two fields for the genotype quality (GQ) and a genotype filter (FT); these fields may be left blank but may be used for post processing and in future versions of Atlas2.

6. References to Atlas2

Atlas2 contributed to the Exon Pilot of the 1000 Genomes Project and is currently contributing to Phase 1 of the 1000 Genomes Project. It is referenced by Marth et al. in "The functional spectrum of low-frequency coding variation" (in press, *Genome Research* 2011).

7. References

Li, H. (2009) Sequence Alignment/Map (SAM) format. SAMtools. Retrieved Jan 20, 2010, from http://samtools.sourceforge.net/SAM1.pdf

Homer N, Nelson SF. Improved variant discovery through local re-alignment of short-read next-generation sequencing data using SRMA. Genome Biol. 2010 Oct 8;11(10):R99

McKenna A, Hanna M, Banks E, Sivachenko A, Cibulskis K, Kernytsky A, Garimella K, Altshuler D, Gabriel S, Daly M, DePristo MA. The Genome Analysis Toolkit: a MapReduce framework for analyzing next-generation DNA sequencing data. Genome Res. 2010 Sep; 20(9):1297-303. Epub 2010 Jul 19.

DePristo, M., Banks, E., Poplin, R., Garimella, K., Maguire, J., Hartl., C., Philippakis, A., del Angel, G., Rivas, M.A, Hanna, M., McKenna, A., Fennell, T. Kernytsky, A., Sivachenko, A, Cibulskis, K., Gabriel, S., Altshuler, D. and Daly, M. A framework for variation discovery and genotyping using next-generation DNA sequencing data. Nature Genetics. 2011 Apr; 43(5):491-498.

Picard. http://picard.sourceforge.net.

8. Change Log

8.1 Atlas-SNP2

a) For Illumina/ 454 platforms

Version 1.3 (08-18-2011)

- Add a new option to call SNP on given regions or by chromosomes
- Change the default maximum coverage for SNP calling to 1024
- For pair-end data, add an option to use insertion size for mapping quality control
- Improve the performance of crossmatch2SAM

Version 1.2 (01-18-2011):

This is a major upgrade of Atlas-SNP2

New features

- one-stop running: take sorted BAM files and reference file as input and output SNP genotypes in VCF format
- use mapping quality score as alignment quality control
- use insertion size as mapping quality control for pair-end re-sequencing data
- more filters are integrated for higher quality SNP calls

Performance

- whole genome SNP calling is doable on a typical PC with 4G memory now. In our test, it can process 1 million reads per 5 minutes for whole exome SNP calling only using one CPU core of Xeon 5520 and 4G memory

Bugs fixed and compatibility

- more robust to alignment errors
- crossmatch2SAM tool is compatible to Ruby 1.9.X now
- a few minor bugs

Version 1.1 (04-26-2010):

- added a heuristics-based genotyping module
- added a column of "numRefReads_afterFilter" in Atlas-SNP2 result file
- revised the header line in Atlas-SNP2 output file to be more explicit
- skipped duplicate reads masked in the BAM files when processing
- added an option for the user to setup the max number of alignments allowed to be piled up at a particular site
- printed more running information and more detailed alignments statistics
- more robust to various alignments errors
- fixed several bugs

Version 1.0 (01-20-2010):

- added Illumina Platform support
- all calculations are now based on required fields of SAM to get maximum compatibility
- added CIGAR and reference sequence test code
- used pileup number to calculate TotalCoverage
- improved performance
- migrated to Ruby 1.9
- many minor improvements

Draft release version 0.1 (12-10-2009):

- initial implementation
- initial support of SAM files

b) For SOLiD platform

Version 1.0 (08-18-2011):

- Major SNP calling model update
- Support GATK base quality re-calibrated BAM by using OQ tags
- Call SNPs only on regions defined in a bed format file
- Output the SNP calls in vcf format directly

Draft release version 0.1 (01-26-2011):

- Initial implementation

8.2 Atlas-Indel2

v0.3.1

- added options to use original base quality
- fixed bug that sometimes returned success exit code when there was a failure
- fixed bug in simple_genotyper that caused samples with exactly 0.05 variant read ratio to be 0/0
- fixed bug in simple genotyper that caused genotypes to occationaly read ./.
- fixed bug in bed_filter that was filtering some on-target reads in very small target regions

v0.3

- updated SOLiD and Illumina models and recalibrated default settings
- Implemented the ability to input a bed file to call only on-target indels
- switched from using z cutoffs to using p cutoffs
- modified 1bp p cutoff to only filter 1bp deletions
- fixed bug where the strand direction filter failed to be enabled

- Added check for proper ruby version
- fixed bug that occasionally allows an indel quality of 110 (max should be 100)
- minor code-structure changes

v0.2.1

- added read_level model and improved site level model for SOLiD data
- adjusted default SOLiD z cutoff to 0.0 (to reflect new model)
- added check for proper ruby version
- minor codes structure changes
- added additional heuristic filter that allows for a stricter z cutoff for 1bp indels, very useful for SOLiD data
- integrated heuristic genotyping –implemented
- fixed bug where Atlas-Indel2 crashes if a BAM chromosome is not in the reference
- now will keep 'chr' in the chromosome label if it is in the BAM
- the depreciated script "Atlas-Indel2-Illum-Exome.rb, has been removed. Please use Atlas-Indel2.rb with the -I flag instead.

v0.2

- Implemented regression model for SOLiD data. You must now specify a regression model with -S or -I.
- Renamed main script to Atlas-Indel.rb.
- Modified Reference sequence class to allow for unsorted reference genomes.
- Added the indel z to the info column of the VCF output (not included after running VCF printer).
- Now echos all settings back onto the command line.
- Fixed a bug that caused loss of precision in the normalized variant square variable of the Illumina site model.
- Fixed a bug in the depth coverage algorithm that caused reads not to be counted in total depth at the deleted sites.
- Fixed the sample columns order to be comaptible with vcfPrinter.
- Removed "x flagged lines skipped" message at end of run.

9. License

Copyright (c) 2011, Human Genome Sequencing Center, Baylor College of Medicine

All rights reserved.

Redistribution and use in source and binary forms, with or without modification, are permitted provided that the following conditions are met:

- Redistributions of source code must retain the above copyright notice, this list of conditions and the following disclaimer.
- Redistributions in binary form must reproduce the above copyright notice, this list of conditions and the following disclaimer in the documentation and/or other materials provided with the distribution.

THIS SOFTWARE IS PROVIDED BY THE COPYRIGHT HOLDERS AND CONTRIBUTORS "AS IS" AND ANY EXPRESS OR IMPLIED WARRANTIES, INCLUDING, BUT NOT LIMITED TO, THE IMPLIED WARRANTIES OF MERCHANTABILITY AND FITNESS FOR A PARTICULAR PURPOSE ARE DISCLAIMED. IN NO EVENT SHALL THE COPYRIGHT HOLDER OR CONTRIBUTORS BE LIABLE FOR ANY DIRECT, INDIRECT, INCIDENTAL, SPECIAL, EXEMPLARY, OR CONSEQUENTIAL DAMAGES (INCLUDING, BUT NOT LIMITED TO, PROCUREMENT OF SUBSTITUTE GOODS OR SERVICES; LOSS OF USE, DATA, OR PROFITS; OR BUSINESS INTERRUPTION) HOWEVER CAUSED AND ON ANY THEORY OF LIABILITY, WHETHER IN CONTRACT, STRICT LIABILITY, OR TORT (INCLUDING NEGLIGENCE OR OTHERWISE) ARISING IN ANY WAY OUT OF THE USE OF THIS SOFTWARE, EVEN IF ADVISED OF THE POSSIBILITY OF SUCH DAMAGE

10. Appendix

A. Explanations of Atlas-SNP2 putative SNP evaluation file

Atlas-SNP2 for Illumina/454 also outputs a middle file containing evaluation of all putative SNPs, each has detailed information described in 18 tab delimited fields. Headers are shown below:

refName<tab>coordinate<tab>refBase<tab>variantBase<tab>oriQual<tab>variantReadCov<tab>numAlternativeReads<tab>totalCoverage<tab>Pr(error)j<tab>Pr(SNP)j<tab>Pr(Sj|error,c)<tab>Pr(Sj|SNP,c)<tab>Prior(error|c)<tab>Prior(SNP|c)<tab>Pr(SNP|Sj,c)j<tab>refEnv<tab>homopolymer<tab>readsInfo

Table 1. Explanations for different fields in the output.

Field Name	Explanation
refName	The name of the reference sequence, for example, chr12.
Coordinate	The physical position of the SNP site on the reference sequence.
refBase	The reference base in that SNP position.
variantBase	The variant base (if there are several variants, it takes the one with largest occurrence).
oriQual	The summation of the phred quality scores of all reads showing the variant base.
variantReadCov_afterFilter	The number of reads after filtered that harbor the same variant base (shown in the variantBase column).
numAlternativeReads_afterFilter	The total number of reads after filtered that differ from the reference sequence on the SNP site.
numRefReads_afterFilter	The number of reads after filtered that harbor reference base
totalCoverage_afterFilter	The total number of reads after filtered at the SNP site.
Pr(error)j	The prior error probability of the locus j when conditioning on variant read coverage.
Pr(SNP)j	The prior SNP probability of the locus j when conditioning on variant read coverage. This entire item is represented by a symbol *Sj*, which stands for signal at locus j.
Pr(Sj|error,c)	This is derived from the probability density distribution of *Sj* of errors at a specific variant read coverage *c.*
Pr(Sj|SNP,c)	This is derived from the probability density distribution of *Sj* of true SNPs at a specific variant read coverage *c.*
Prior(error|c)	Prior estimation of the substitution error rate when conditioning on variant read coverage.
Prior(SNP|c)	Prior estimation of the substitution SNP rate when conditioning on variant read coverage.
Pr(SNP|Sj,c)j	The Posterior SNP probability of the locus j when signal is Sj at a specific variant read coverage *c.*
refEnv	The reference sequence of a 13-bp window centered on the SNP site.
Homopolymer	The size of the longest homopolymer within a 13-bp window centered on the SNP base on the reference sequence (legacy column, only useful for manual inspection).
*readsInfo**	Information about the reads that harbor the same variant base.

*The field “readsInfo” contains a list of semicolon-separated strings as shown in the following example. Each of the strings contains the related information of the respective variant read. The following example shows the format of one of the strings in field “readsInfo”. Table 2 gives the detailed explanation about each element within the string shown below as an example.

T(15)EIXH2IB02H9BM7(16)(235.0/272)+taacccTaaccta(0.38/1.92)(0/272)snp(0.861);

Table 2. Explanations for “readsInfo”

String name	Explanation
T	The variant base
15	Raw phred-like quality score
EIXH2IB02H9BM7	The read name
16	The distance of the variant base to right end of the read
235.0/272	Smith-waterman score of match / The queried size on the reads
+	The read direction of strand
taacccTaaccta	The variant read sequence of a 13-bp window centered on the SNP site.
0.38/1.92	%subsitutions in matching region /%indels in matching region
0/272	The status of “NQS pass” / the length of the reads
Snp	This variant base is a substitution from the reference sequence to the read sequence
0.861	The prior estimated error probability of this variant base i, Pr(error)i
