# Supplementary material for: An integrative variant analysis suite for whole exome next-generation sequencing data
Source: BMC Bioinformatics. 2012 Jan 12;13:8. doi: 10.1186/1471-2105-13-8 (PMC3292476; doi:10.1186/1471-2105-13-8)
Supplement: Additional file 3 — The Atlas2 Suite version 1.0. [file 1471-2105-13-8-S3.BZ2 › Atlas2_v1.0/Atlas-SNP2/Auxiliary/Additional 454 Documentation.pdf]

A mapping pipeline for 454 data

1. Introduction
The whole workflow includes three steps: "Atlas-SNP-mapper", "Atlas-SNP-splitter" and "Atlas-SNP-SAM" (Figure S1.1)

"Atlas-SNP-mapper" is a wrapper that takes the reference genomic sequence, the NGS reads (including sequence fasta and the quality files) as the inputs, and generates mapping results in *cross_match* output format.

"Atlas-SNP-splitter" next takes the outputs and NGS reads quality fasta files (that need to be partitioned) as its inputs. After splitting, its outputs are a series of smaller batches. This module is designed for the resource management purpose when NGS data size is large.

"Atlas-SNP-SAM" takes the outputs, read file and the read quality files and reference sequence file as the inputs to generate alignment files that are in SAM format. This is only a simple file reformatting step.

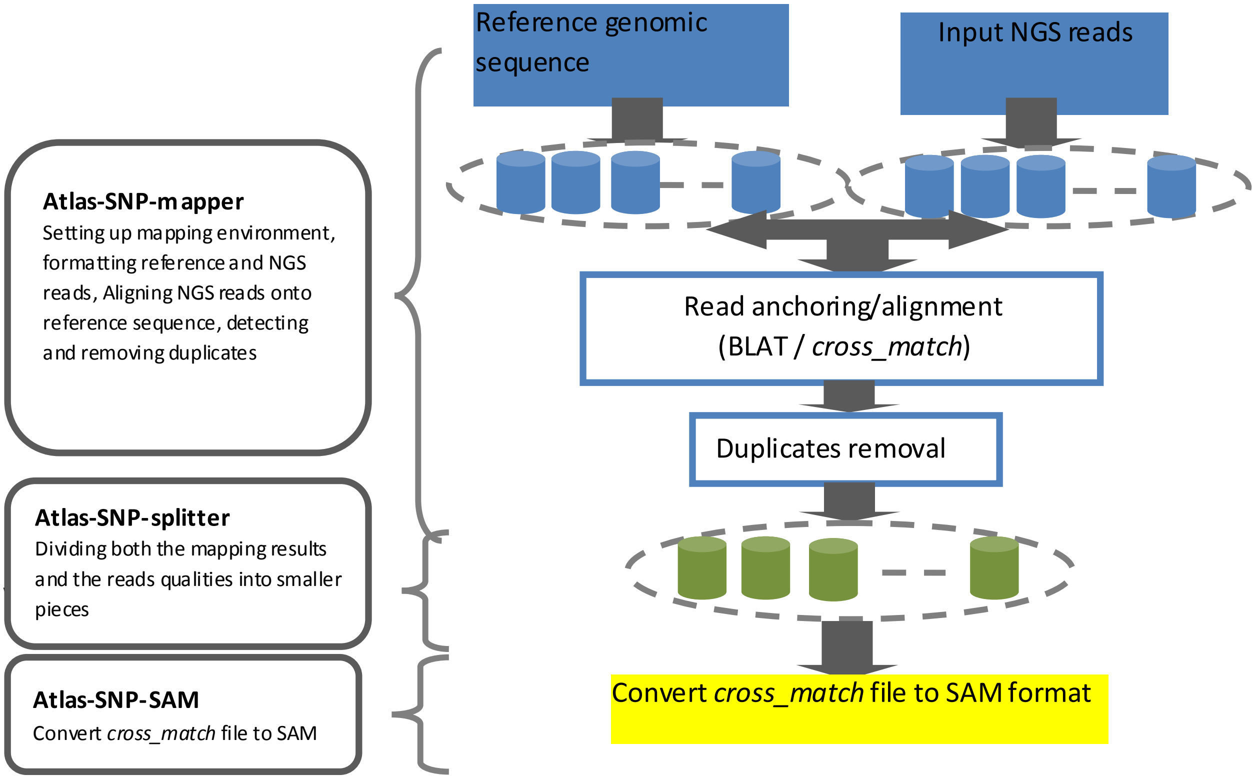

Figure S1.1 The overall "454 raw reads->BLAT/Cross-match->SAM" workflow.

Step 1: Atlas-SNP-mapper (Figure S1.2)

Atlas-SNP-mapper splits reference genomic sequences to smaller regions with the size ranging from 10Kb to 10Mb per region. It also divides NGS read files and read quality files into smaller pieces, ranging from 5Mb to 10Mb in size for the purpose of computational resource management. It uses the divided pieces from both the genomic reference sequence and NGS reads as inputs to attempt to anchor and align all the NGS sequences onto the reference sequence. A few further steps such as "duplicates removal" can be used to remove experimental artifacts from sequencing.

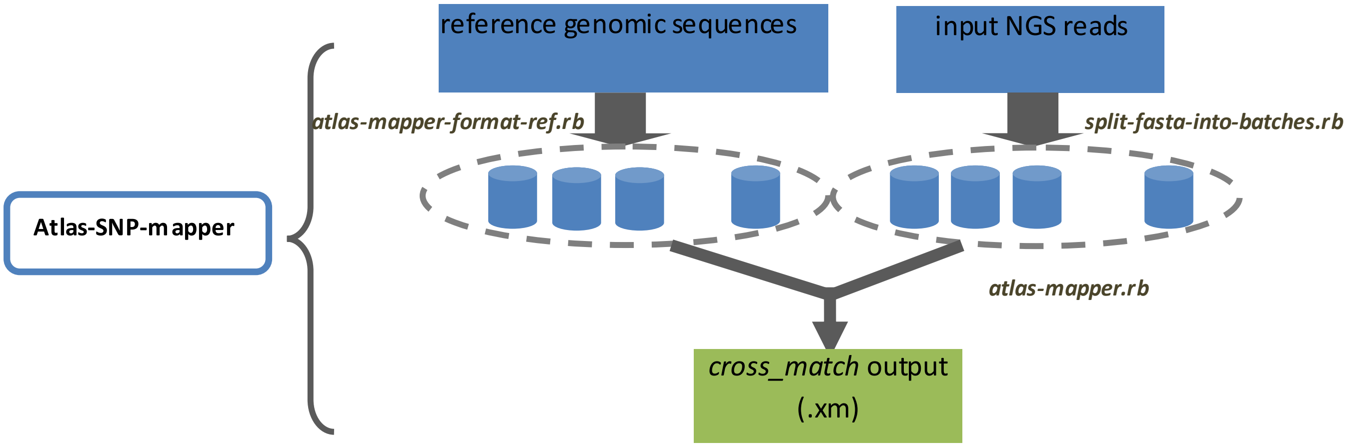

Figure S1.2 Atlas-SNP-mapper

Step 2 : Atas-SNP-splitter (Figure S1.3)

Atlas-SNP-splitter splits mapping results and respective quality files of the successfully mapped reads into smaller batches. The program first splits the mapping result by chromosomes into batches, and further splits the batches within each chromosome into smaller batches that roughly have the same size. Next, two more steps are applied to categorize the successfully mapped reads into batches by looking up the read name from the original mapping batches.

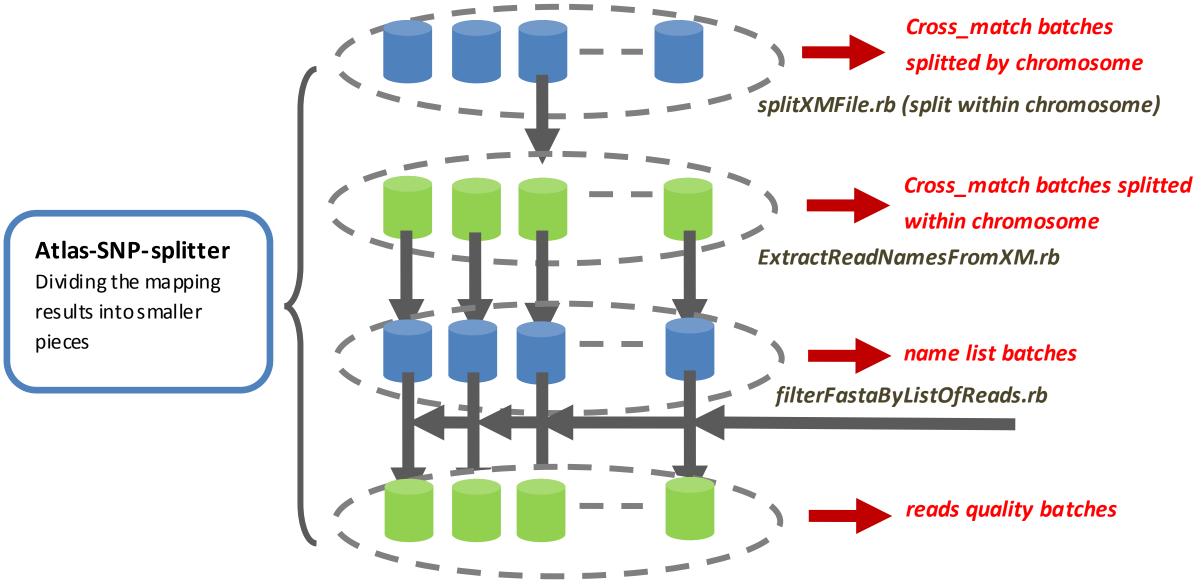

Figure S1.3 Atlas-SNP-splitter

Step 3: crossmatch2SAM (Figure S1.4)

crossmatch2SAM.rb is designed to convert *cross_match* format alignment files into SAM format.

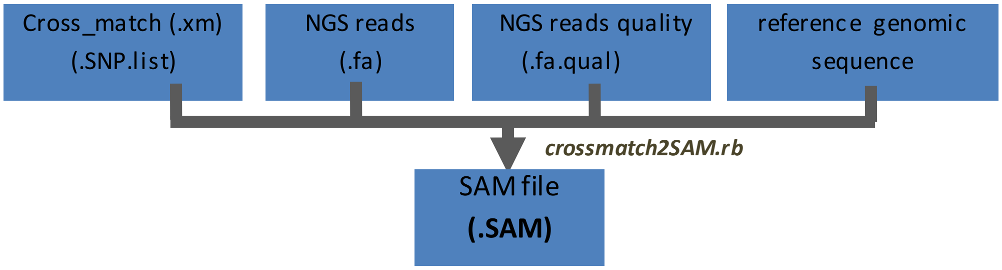

Figure S1.4 cross_match2SAM

2. Usage

2.1 Atlas-SNP-mapper

ruby atlas-mapper-format-ref.rb [-r reference] [-l length of each piece] [-f frequency Cutoff of 11mer] [--help]

“atlas-mapper-format-ref.rb” aims to divide the reference genome into pieces to meet the computational requirements when running “BLAT” and “*cross_match*” respectively. The output will be a newly created directory named “*referenceName*.Environment”, under which reference fragments, the fragment index and the relative information about the splitting are placed. Under this directory, there are two sub directories: “ref-pieces” and “ref-divisions”. The longer divided reference pieces (900Mb in size) are placed under the sub directory “ref-pieces”, which will actually be used for “BLAT” to anchor reads back to respective local regions; the shorter reference pieces (100Kb in size) are stored under the sub directory named as “ref-divisions”, which are used to precisely compare the bases of read sequences with those on the reference sequence using “*cross_match*”. This program takes two required parameters, “--reference” (“-r”), which introduces the reference sequence, and “--length” (“-l”) which is associated with the size of bases each reference piece may contain.

<u>Options:</u>

-r FILE reference genome (required)
-l INT bases a splitted reference piece may contain. With default value 1000000 (required)
-f FLT the cutoff of 11-mer frequency deemed as over-represented in the reference so BLAT will ignore it during seeding step, with default value 1024. 1024 is optimized for mammalian genomes. 100~200 is best for smaller or less complex genomes.
-h usage information

ruby atlas-mapper.rb [-r reference] [-q reads fasta file] [-m minScore] [-c cutoff] [-b blat] [-c crossMatch] [-n oneOff] [-i minIdentity] [-t minMatchRatio] [-l mastLevel] [-z xmOnly] [-a blatOnly] [-e noParse] [-s short]

“atlas-mapper.rb” takes two required parameters. The first is “--reference” (“-r”), which must be followed by the reference sequence name. The second is “--query” (“-q”) which is associated with the NGS reads name. By running this wrapping program, users don’t have to take efforts in any intermediate steps, such as parsing “BLAT” results, picking the best hits and format conversion between the two tools. Also, the program provides flexibility that allows users to control the pathway. For example, once the option “-z” is appended, the program will only run cross_match, the same will happen for the option “-a” for running “BLAT” only.

Options:

-r	FILE	reference genome (required)
-q	FILE	reads fasta file (required)
-m	INT	parameter of “crossmatch”, minimum score with default value 30
-c	FLT	% for the best hit cutoff with default value 0.99
-b		inferring blat executive file
-c		inferring crossmatch executive file
-n		oneOff set to run “BLAT” with default value 0
-i	INT	minimum identities set when running “BLAT” with default value 90
-t	FLT	minimum match ratio when grouping the best hit reads generated by “BLAT” with default value 0.85. Definition: matched bases / reads length
-l	INT	mask level set when running “crossmatch” with default value 20
-z		causing program to run “crossmatch” only
-a		causing program to run “BLAT” only
-e		causing program to stop right after “BLAT” without parse out the best hit reads.
-s		option for aligning short reads using “crossmatch”

Note: for more details on the parameter usage and output format, please refer to the “*cross_match*” and “BLAT” documentation.

2.2 Atlas-SNP-splitter

ruby splitXMFile.rb [-x crossmatch file] [-C split by chromosome] [-n number of parts of the input with mapping on genomic regions of the same size] [-o output file root] [-h help]

“splitXMFile.rb” is a flexible data splitting tool, designed especially for splitting the *cross_match* file to meet the computational requirements for running “Atlas-SNP-core”. Besides the regular input *cross_match* file introduced by the parameter “--xmFile” (“-x”), the tool provides other two important parameters —“--splitByChrom” (“-C”) and “--numberOfParts” (“-n”). If “-C” is appended, the *cross_match* file will be divided by chromosomes. “-C” is a MUST for large dataset with multiple reference regions like the human genome. Another option “--numberOfParts” (“-n”), is associated with a number defined by users. This option permits the program to further divide the *cross_match* file into the size determined by users. We have used “-n 20” for processing the human genome, for example. Please note that the option “-C” must be used whenever “-n” is used.

Options:

-x	FILE	crossmatch file (required)
-C		causing the program to split crossmatch file by chromosome
-n	INT	number of parts that the crossmatch file will be divided within one chromosome
-o	STR	output file root (required)
-h		usage information

ruby extractReadNamesFromXM.rb [crossmatch file] [output file]

"extractReadNamesFromXM.rb" extracts all the read names from the *cross_match* files and places a list of these names into an output file. This name list is used as a small trace id database to be queried by the program to pick the corresponding reads. The program takes two arguments, the *cross_match* file name and an output file name.

ruby filterFastaByListOfReads.rb [-f fasta file of reads] [-l file containing the list of reads to be selected] [-o outputPrefix] [-c fasta file chunk size, in number of sequences]

"filterFastaByListOfReads.rb" is used to filter those NGS reads by choosing only the reads in the trace ids database created by "extractReadNamesFromXM.rb". By only considering the aligned reads, the time and memory usage for running "atlasNQSPASS.rb" can be largely reduced. The program takes three required parameters: "--fastaFiles" ("-f") for the reads fasta file; "--readListFile" ("-l") that is followed by the "trace id database" file created by "extractReadNamesFromXM.rb"; and "--outFile" ("-o") that specifies the output file name. The program will generate a .gz fasta file containing only the sequences of the aligned reads.

<u>Options:</u>

-f	FILE	reads fasta file (required)
-l	STR	the output file of "extractReadNamesFromXM.rb" (required)
-o	STR	the output file prefix (required)
-c	INT	fasta file chunk size (optional)
-h		usage information

2.3 Atlas-SNP-SAM

ruby crossmatch2SAM.rb [-i cross_match] [-o output file] [-r read sequence] [-q read quality file] [-f reference] [-o output]

For each aligned read, the program parses out the mismatched substitutions and indels from *cross_match* results. In the meantime, the program keeps querying subsequences from both reference sequence and raw reads file (sequence and quality file). The program takes the cross_match file ("-i"), read sequences ("-r"), the read quality file ("-q") and the reference sequence file ("-f") as input files, and then produce a SAM format mapping file as the output file (see Table4).

<u>Option</u>

-i	FILE	cross_match file (required)
-o	STR	output file indicated by users (required)
-r	FILE	NGS read sequence file (required)
-q	FILE	NGS read quality file (required)
-f	FILE	reference sequence file (required)
-h		usage information
